# Micro-Satellite Systems Design, Integration, and Flight

**DOI:** 10.3390/mi15040455

**Published:** 2024-03-28

**Authors:** Philip Naumann, Timothy Sands

**Affiliations:** 1 Systems Engineering Program, Cornell University, Ithaca, NY 14853, USA; pn246@cornell.edu; 2 Department of Mechanical Engineering (SCPD), Stanford University, Stanford, CA 94305, USA

**Keywords:** systems engineering, MBSE, RF communication, GFSK, CDMA, forward error correction, matched filtering, TI-RTOS, RTL-SDR, TinyGS, controller optimization controller modeling, controller verification and validation, Kane damper, PD controller, IMU tuning

## Abstract

Within the past decade, the aerospace engineering industry has evolved beyond the constraints of using single, large, custom satellites. Due to the increased reliability and robustness of commercial, off-the-shelf printed circuit board components, missions have instead transitioned towards deploying swarms of smaller satellites. Such an approach significantly decreases the mission cost by reducing custom engineering and deployment expenses. Nanosatellites can be quickly developed with a more modular design at lower risk. The Alpha mission at the Cornell University Space Systems Studio is fabricated in this manner. However, for the purpose of development, the initial proof of concept included a two-satellite system. The manuscript will discuss system engineering approaches used to model and mature the design of the pilot satellite. The two systems that will be primarily focused on are the attitude control system of the carrier nanosatellite and the radio frequency communications on the excreted femto-satellites. Milestones achieved include ChipSat to ChipSat communication, ChipSat to ground station communication, packet creation, error correction, appending a preamble, and filtering the signal. Other achievements include controller traceability/verification and validation, software rigidity tests, hardware endurance testing, Kane damper, and inertial measurement unit tuning. These developments matured the technological readiness level (TRL) of systems in preparation for satellite deployment.

## 1. Introduction

**Figure 1:**
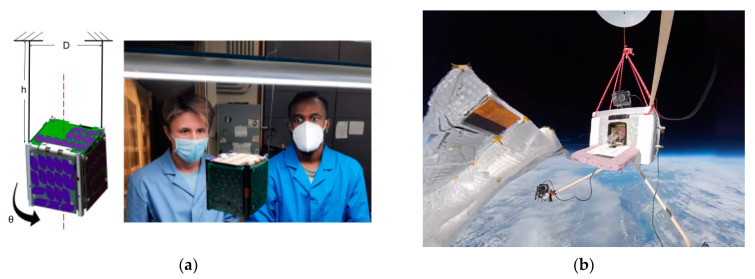
(**a**) Moment of inertia testing for CubeSat attitude control system development; (**b**) balloon test to validate ChipSat long-range radio frequency capabilities.

The development of a nanosatellite as a carrier for a fleet of light sail-propelled picosatellites (ChipSats) is depicted in Figure 1.

Satellite development has historically been a complex and expensive venture, accessible only to government agencies and large industry at a high financial and technological barrier of entry. NASA’s space shuttle had a cost of about USD 1.5 billion to launch 27,500 kilograms into low-earth orbit, or USD 54,500 per kilogram [1]. Meanwhile, commercial launches have reduced the cost of low-earth orbit twenty-folds. There is an increasing urgency to expand horizons in space, not only for scientific knowledge and exploration but also for interplanetary travel and access to natural resources.

### 1.1. Background on Satellite Development

In response to the rampant development of electronic systems in the early 21st century [2], a gateway to cheaper satellite development has emerged, allowing for competition in the commercial space. Development within the areas of microchips, semiconductors, and batteries has paved the way for cheaper, lighter, and more powerful cyberinfrastructures. Complex satellites are now being fabricated at the nano/picosatellite scale. Additionally, satellite designs are becoming more modular. Commercial off-the-shelf (COTS) components have replaced custom-developed ones, allowing for mature, modular technologies and faster turnaround times.

#### Background of the Alpha Mission

The manuscript will examine systems design and analysis of the Alpha mission developed at the Cornell Space Systems Studio. The mission aims to be a proof of the concept of interstellar travel, furthering the maturity of (1) a CubeSat satellite deployer, (2) an autonomous picosatellite [3], and (3) light sail technology [4]. These technologies would parallel techniques proposed by the Breakthrough Starshot initiative. The CubeSat approach was a vital medium for this mission. Due to its modular and versatile chassis, a CubeSat was used as the capsule of our payload. Using the standardized CubeSat structure will simplify replication and further development for other developers [5]. In addition, other CubeSat missions [6,7] could re-use the approaches outlined in this manuscript. Most importantly, the CubeSat was used to mimic the approach of Breakthrough Starshot [8]. Breakthrough Starshot proposes the first solution for space travel into another solar system [9]. The idea theorizes accelerating a diminutive payload (with a mass of about a gram) to a fifth of the speed of light propelled by high-power lasers reflecting off a light sail [10]. According to the theory, the tiny payload would be accelerated within seconds. With the payload pointed at Alpha Centauri (the closest star system outside of our solar system) at 4.37, it could be possible that this distance could be reached within 25 years. To minimize the risk of failure, thousands of payloads would be deployed with the hope of a few being successfully received by the Alpha Centauri solar system. The purpose of the Alpha mission is to successfully deploy and stabilize a single payload light sail system [11]. For this to happen, the carrier CubeSat detumbles and stabilizes itself, successfully ejects the light sail payload, and the payload must power up and communicate with the ground station [12,13].

### 1.2. Background on Subsystems Covered in This Manuscript

The manuscript will review the two most influential systems responsible for meeting the mission goal, namely, the stabilization of the CubeSat and the radio frequency (RF) communication infrastructure between the picosatellite (ChipSat) and ground station.

The first half of the manuscript will discuss the use of RF communication for robust, long-range, and low-power transmissions. For the Alpha project, the picosatellite payload was developed by Dr. Van Hunter Adams [14,15], (previously at Cornell). The satellite is printed on a thin Kapton substrate [16,17] and only has a solar panel, processor, IMU, GPS, light sensor, and RF transceiver. The satellite is named ChipSat.

The second half of the manuscript will discuss the ACS of a 1U CubeSat. This development was built off the progress of Armin [18] and Carabellese [19] (previously at Cornell). For the Alpha mission, a CubeSat is used to transport our payload because of the affordability of the satellite type [20] and the simplicity the satellite structure offers. The one-unit (1U) CubeSat is a 10 cm by 10 cm by 11.35 cm sized satellite [21] with small rectangular rails on each of the corners. CubeSats can exist in several multiples of the one-unit size up to 12U [22]. The modular size standard of the CubeSat is chosen such that several of these satellites can be easily loaded onto a canisterized satellite dispenser (CSD) [23,24]. The CDS dispenser is spring-loaded and fastened to the payload of a launch vehicle [25]. When activated, the CSD will eject the payloads into low-earth orbit (LEO).

#### Systems Analysis Approach

**Figure 2:**
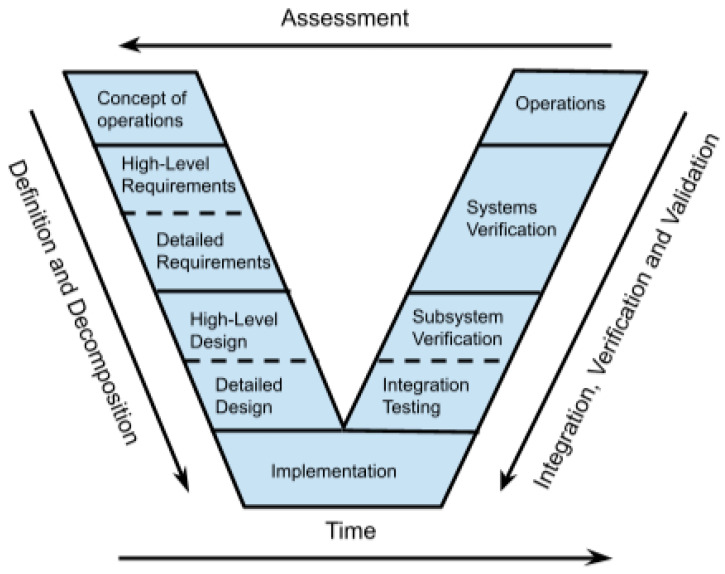
The systems engineering “Vee Diagram”.

The manuscript will use a model-based systems engineering (MBSE) approach [26,27,28] to analyze and design both the CubeSat and the ChipSat. The MBSE approach maintains traceability throughout the satellite’s life cycle [29]. The design process can be shown by the systems engineering Vee diagram [30], which is a flow chart of the product life cycle. The left side of the Vee diagram shows how the design process goes from a high-level concept to specific component-level requirements. Then, the right side of the Vee diagram shows the integration process. On the right side, the system is assembled from component to working prototype through validation and verification methods. Expressed briefly, a product is designed from the top down but tested from the bottom up. The Vee approach makes sure that the design remains consistent with the original problem description. It also guarantees final functionality. Testing begins at the component level, so that functionality is tracked up to the final acceptance testing. Below, in Figure 2, is a visual representation of the Vee diagram used in the manuscript.

The manuscript will use the Vee diagram process. Section 2 starts with modeling the mission systems. The satellite systems are described and then broken down (top-down from concept design to the component level).

Section 3 and Section 4 track the ChipSat and CubeSat development. The satellite requirements are stated and then followed bottom-up through verification and validation tests.

## 2. Systems Modeling of the CubeSat

### 2.1. Function-Centric Models

The design process [31,32] began with a context diagram of the CubeSat nanosatellite. The context diagram shows the external and internal interfaces to track resource usage from start to finish and set clear interface boundaries. The modeling approach followed the systems models created by George E. Mobus and Michael C. Kalton in the book “Principles of Systems Science” [33].

**Figure 3:**
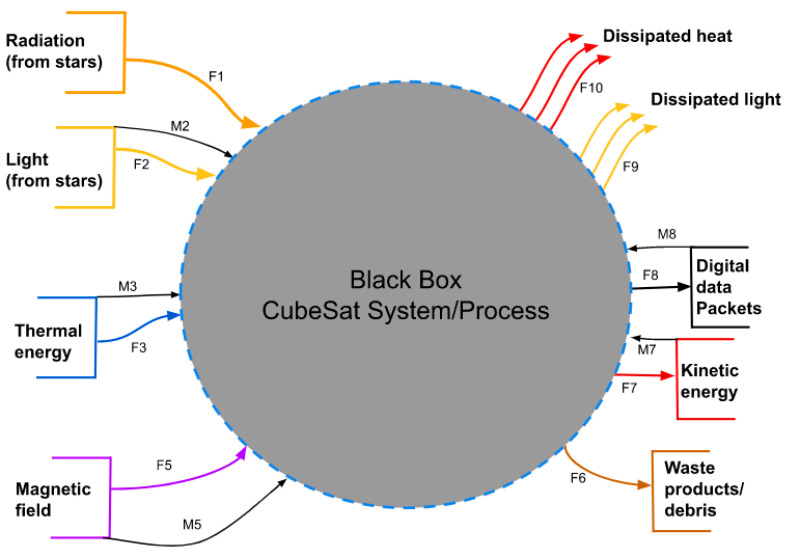
Black box diagram (George E. Mobus and Michael C. Kalton, p. 604 [33]).

A concept diagram, as defined by George E. Mobus, identifies the sources, sinks, stocks, and interfaces of a system. These were first found externally. Figure 3 presents the “Black Box” of the CubeSat system. Here, all of the system inputs and outputs can be easily seen. The inputs are labeled as sources, as shown on the left of the diagram. The sinks are shown going out of the system to the right. In equilibrium, there is conservation of mass and energy across the system. See Figure 3.

The function that a resource flow goes through is represented by the F values in Figure 3. The M indicates a movement of data inside the function. Table 1 explains what the functions in Figure 3 mean.

**Figure 4:**
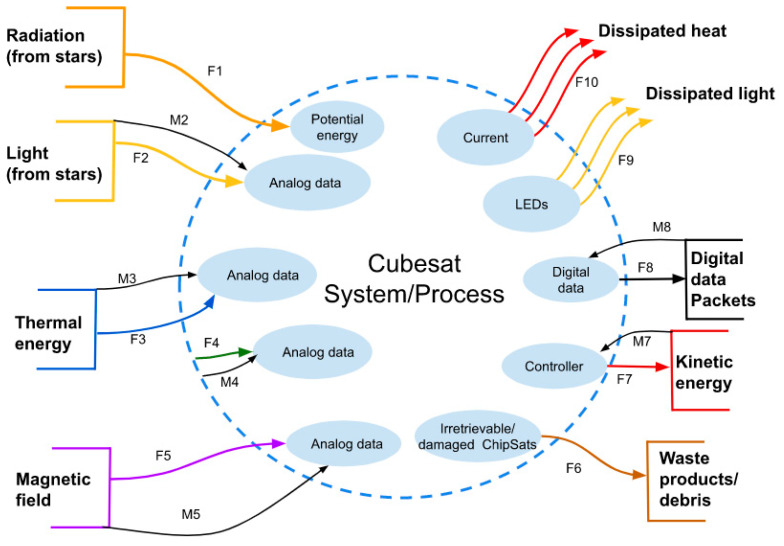
Subsystem black box diagram (George E. Mobus Michael C. Kalton, p. 607 [33]).

A useful way to analyze the system is to divide it into smaller parts and see how they influence each other. The products that are produced in the “Black Box” can be by-products, waste products, catalysts, or reaction intermediates that help achieve the intended functional requirements. By understanding the interactions between subsystems and their products both inside and outside of the system, it was possible to improve the interfaces within the software more effectively. See Figure 4.

The IMU function that the system uses is shown in Table 2. The IMU location does not depend on any input from the environment, so there is no outside source.

**Figure 5:**
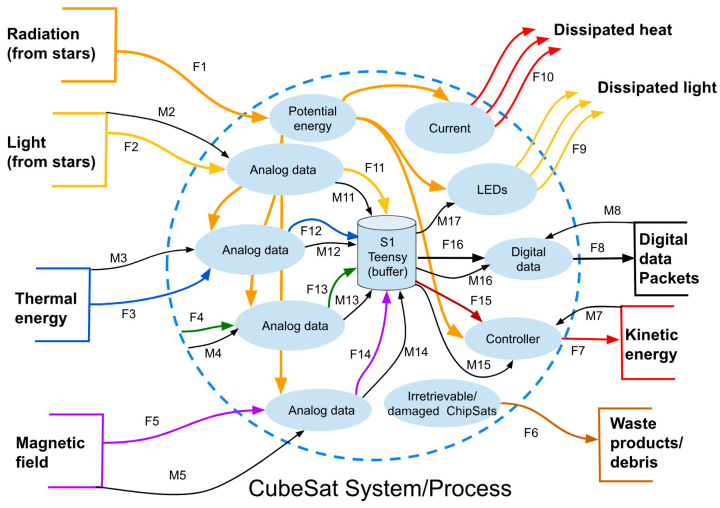
Process diagram (George E. Mobus Michael C. Kalton, p. 609 [33]).

The model can also be improved to show how energy and information are transferred within the system. The internal interface diagram shows the flow, stocks, buffers, amplifiers, and valves of the model. Moreover, an internal network can map the resources and track the flows from where they begin to where they end. Then, mass and energy continuity can be followed. The internal interfaces show a complete view of every function that the CubeSat needs to do to move from its inputs to it outputs. In the center of Figure 5, S1 is visible. S1 is the buffer memory of the Teensy microcontroller. The data collected are sent to the buffer and then changed into digital data packets that are then moved through the transceiver. See Figure 5.

Table 3 defines the processes in Figure 5 that are part of the satellite system. They handle the processing and setting up of the data packets.

These models enabled the representation of the exchanges with the external environment and the movement of resources inside.

### 2.2. Network Models

Figure 2, Figure 3 and Figure 4 show models that focus on functions; a model that shows subsystems at the individual level is also needed. A network model would be a better way to analyze a system at the component level because it reveals the interface between the subsystems.

Using network theory made it possible to see more clearly how the components interact. There are different network models that could model the CubeSat system. Even though the subsystems could be modeled as clusters, they all connect to the processor in a broader sense. So, for this manuscript, a central network model is enough.

**Figure 6:**
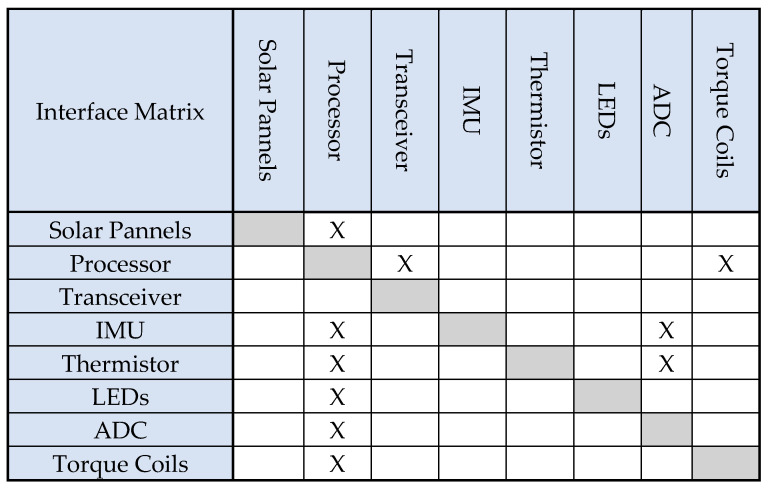
Interface matrix.

To make a network diagram, the context of each component is recorded. A common way to make a network diagram is to list all of the components of a subsystem and make an interface matrix. A more general system interface matrix was made for simplicity. See Figure 6 below.

The relationships in Figure 6 also have a direction. Each cell with an X marks an interaction between two subsystems. The side of the diagonal that an entry is on indicates the direction of the interaction. An X means that a flow starts from the subsystem in the row and ends at the subsystem in the matching column.

**Figure 7:**
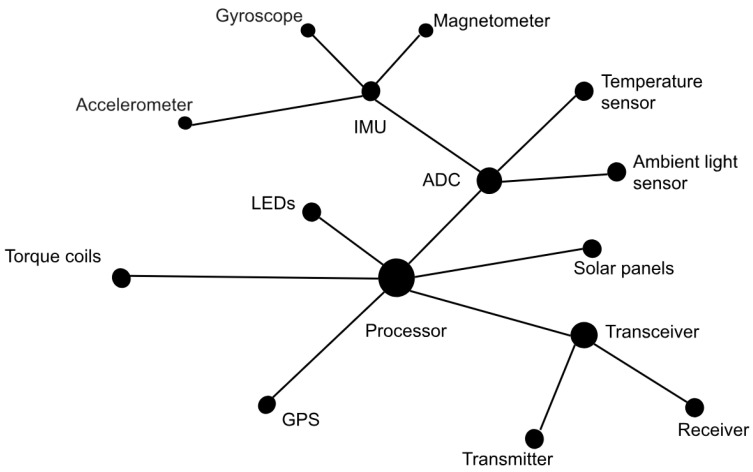
Network diagram.

Based on the information in Figure 6, the network diagram can be derived from the interface matrix. Figure 6 shows the basic structure of the network in CubeSat. The network in CubeSat can show how subcomponents that need an interface are connected. See Figure 7.

**Figure 8:**
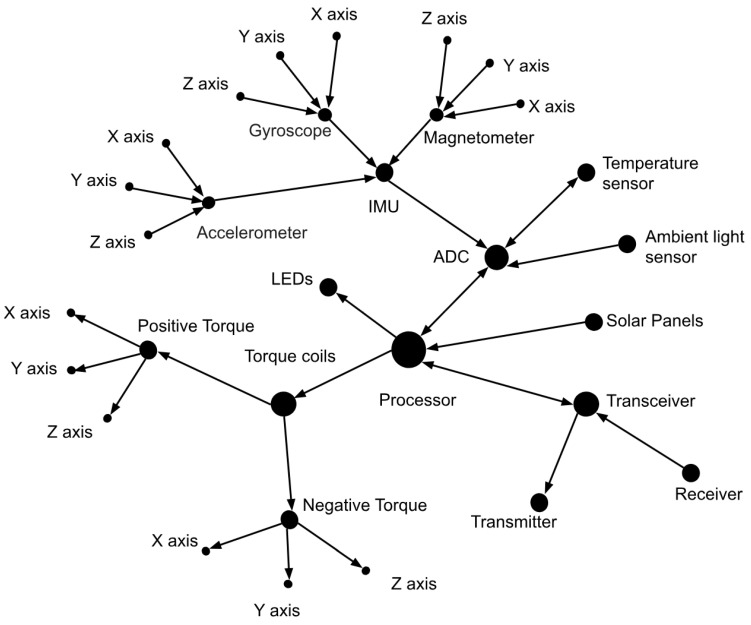
Full network diagram.

The system becomes clearer when the subsystems are separated and the directions are added to Figure 7. Figure 8 displays the five sensors, the torque coils, solar panels, and RF transceiver that the CubeSat has. This helps to see how data move in the system. Refer to Figure 8 below.

Figure 8 shows each value that each of the sensors recorded. This breakdown shows how the data points are recorded by the Teensy 3.5 microcontroller in real time. Each data packet in the network has 17 different data points

### 2.3. Flight Assembly

**Figure 9:**
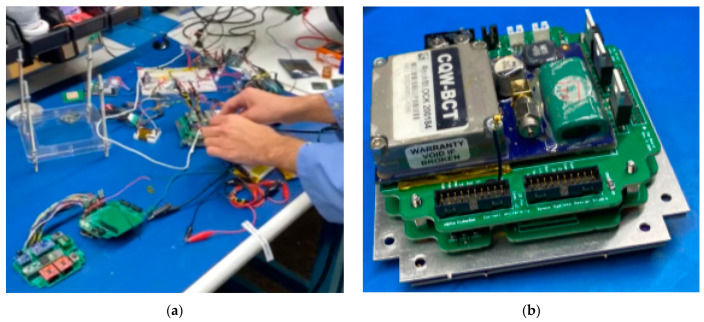
(**a**) Integration testing flight hardware; (**b**) flight-ready electronics aboard the CubeSat.

To take a systems perspective of the CubeSat’s hardware and assembly, the Engineering Development Unit (EDU) satellite was constructed. The EDU was built to perform verification and validation testing, as shown on the right side of the Vee diagram. A complete version (EDU) of the satellite was made. The EDU version was the same as the flight version. However, it would not go to space; it only serves as the standard for the flight satellite. See Figure 9 below.

Some of the components used include the following:

- Teensy 3.5 flight computer microcontroller.
- RockBLOCK radio transceiver (Iridium network).
- Adafruit LSM9DS1 IMU.
- Two SparkFun TB6612FNG dual Motor Drivers.
- INA169 Analog DC Current Sensor Breakout Board.
- 5 V regulator.
- 5 V Reg cam.

The documentation that was made while putting together the EDU version helped to make the Flight Unit CubeSat. Also, the work instruction that was created was used as a reference for other modular CubeSat missions.

## 3. ChipSat RTOS and RF Development

This section discusses the testing and modification of the RF system on the ChipSat picosatellites, one of the systems that was worked on. The system level requirements for Alpha are shown in Table 4 below.

### 3.1. Testing RF on the TI Launchpads

To transmit the sensor readings to be recorded, EM communication is needed because the ChipSats are not going to be retrieved. This fulfills requirement 1.0 (see Table 4), which was achieved using RF communication.

The TI launchpad CC1310 was used as a testing device when developing the RF transmission code since it has the same microcontroller [34] as the ChipSats. The TI boards made the testing setup easier because the ChipSats had to be programmed through the launchpad. Also, it reduced the risk of harming the ChipSats if something went wrong at the beginning.

**Figure 10:**
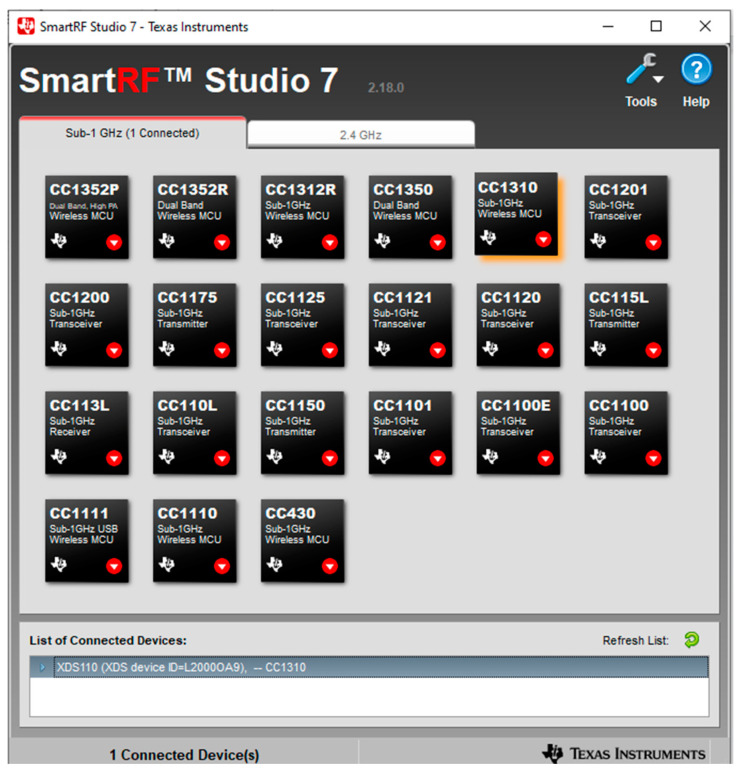
Connecting to TI launchpads on SmartRF Studio 7.

The functionality of the TI launchpads and the speed, range, power, and filtering abilities of their transmissions could be checked and tested using SmartRF Studio version 2.18. Two TI launchpad CC1310s [35] were used with SmartRF Studio 7 for these tests. After installing and opening SmartRF Studio 7, the following screen will show up. See Figure 10.

**Figure 11:**
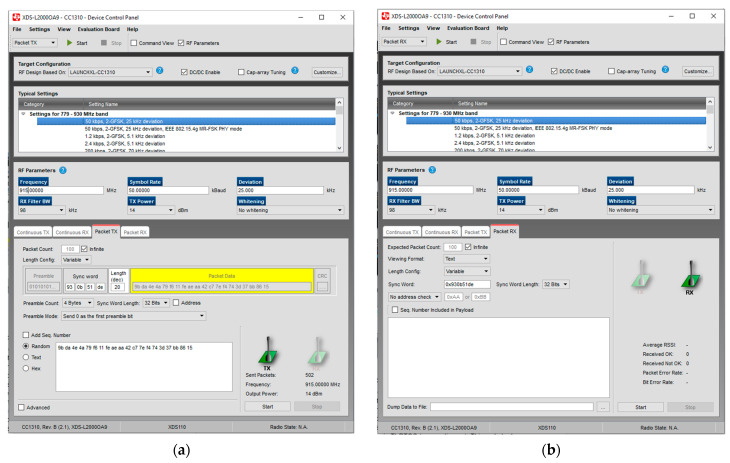
(**a**) Configuring the PacketTX in SmartRF Studio 7. (**b**) Configuring the PacketRX in SmartRF Studio 7.

Figure 10 shows a list of connected devices in the bottom command window. It is recommended not to plug in both TI launchpads to the computer at first since they might be hard to tell apart. Instead, plugging the boards in one by one would let the user decide which board will be the transmitter and which one will be the receiver (marking them might help). By double-clicking on the device from the list of connected devices, the device control panel could be opened. The transmitter should use the packet TX mode. The transmitter should be set up as shown in the following screenshot. See Figure 11. The receiver should use the packet RX mode. It would be useful to have two separate windows open so both the RX and TX screens can be viewed at the same time. Make sure the sync words are the same for both the RX and the TX. Suitable settings for the RX receiver can be seen in the screenshot, see Figure 11 below.

Both the transmitter and receiver have a start button at the top of the screen that needs to be pressed. The receiver will wait for the code word. So, it is necessary to start the receiver before the transmitter if one wants to obtain all the packets. This interface allows us to check the transmitting speeds, range, and power. As we expected, the launchpads sent signals well at nine-hundred and fifteen megahertz. The highest power was around fourteen decibels, compared to twenty-five milliwatts of transmitting power. The transmission distance was about 400 m.

### 3.2. Switching to a Code-Based RTOS Structure

Code Composer Studio (CCS), a Texas Instruments coding platform that uses C++, was used to program the TI launchpads.

Writing RF code without an operating system (OS) or scheduler was not feasible because it depends on a sequential order of code execution. It would have been impossible to give tasks the appropriate priority. For instance, if you had two tasks, stabilize and transmit, you would need to stabilize first before transmitting. Without an OS or scheduler, you would have to place one task above the other in the code sequence. But if these tasks run at different times, sharing resources becomes complex. Therefore, an OS, or scheduler, is needed. The OS, or scheduler, assigns a priority to each task and schedules them accordingly. A common type of OS or scheduler is the real-time OS, or RTOS. In CCS, the RTOS is called TI-RTOS. The TI-RTOS scheduler has four main levels of priority. If a higher-priority operation is scheduled during any operation, the OS or scheduler will pause the current operation and switch to the higher-priority one. The highest level of priority is the hardware interrupts (hwis). The priority among the hardware interrupts is determined by the semiconductor used. When a hwi finishes executing, the scheduler will resume the next highest hwi.

If there are no more hwis, the scheduler will move to the next level: software interrupts (swis). The swis execute in a similar way. However, the priority is set in the software. There are 32 different levels of priority available.

If there are no hwis and swis, the scheduler moves to the task thread. The task thread is where the normal tasks are performed. Normal tasks would include acquiring GPS data, IMU data, or RF tasks. There are also 32 levels of priority in the task thread.

**Figure 12:**
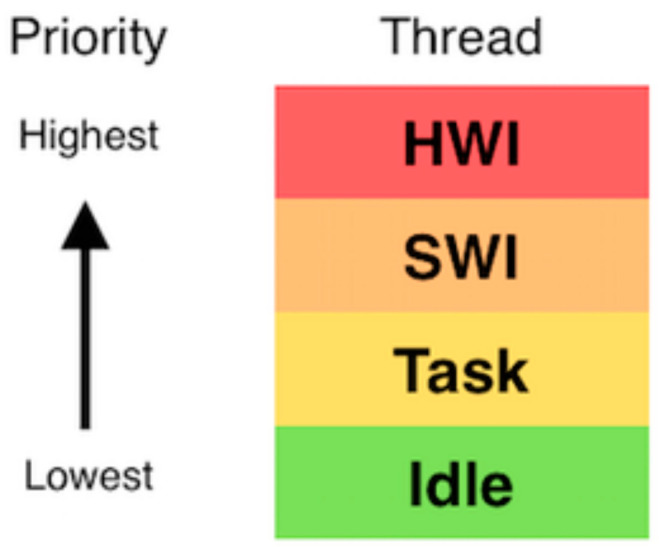
TI-RTOS kernel priority levels. Image Credit: TI [34].

The lowest level is the idle mode. The idle mode only happens when there are no interruptions or tasks. In this mode, there is no priority level, and low-level background computation is performed. The idle mode is used to keep track of time, run low-priority background programs, and check for hwis, swis, or tasks. Mainly, the idle mode is used to save energy. Figure 12 below shows a summary of the scheduler priority levels.

**Figure 13:**
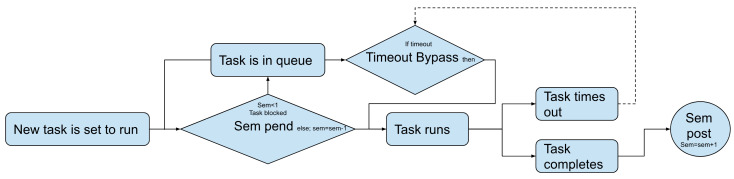
Semaphore process flow.

The task mode also has its own scheduler. The internal scheduler lets programs run at the same time but limits resources or time-sets for data collection. A semaphore is used to manage tasks properly. The semaphore keeps track of how many tasks are performed using a resource, controls the use of the resource, and keeps a queue. There are two types of semaphores: a binary semaphore and a counting semaphore. The binary semaphore only lets one task run at once. When the binary semaphore is equal to 1, the resource is free. After a task takes the resource, a semaphore pend is posted. The pend subtracts one from the semaphore, making it zero, and blocks the channel. When the task finishes using the resource, a semaphore post is posted. The post restores the semaphore count, making the resource available again. The ChipSat uses a counting semaphore. The counting semaphore works like the binary semaphore, but it allows for *n* different tasks to access a resource at once. The semaphore is initialized at *n*. Tasks can post and pend to the semaphore in the same way as in a binary semaphore, but when the semaphore is less than 1, the resource is blocked. See the flowchart below in Figure 13. The counting semaphore allows for a task to time out. A message is then sent to timeout bypass to skip the next task in the queue. The timeout bypass is shown by the dotted line in Figure 13.

**Figure 14:**
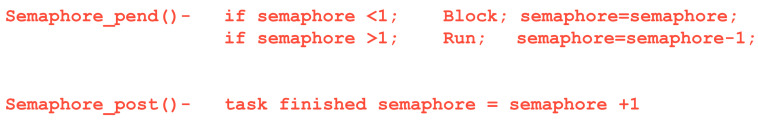
TI-RTOS semaphore APIs.

The following APIs are for posting and pending to a semaphore in the TI-RTOS kernel. See Figure 14 below.

The RTOS structure enables smooth transitions between sensors and actuators on the ChipSat.

### 3.3. Implementing RF Communications on TI Launchpad Using CCS

The next goal was to achieve communication between two ChipSats using radio frequency (RF). Code Composer Studio (CCS) was the tool used to program the RF tasks. The Alpha mission only required transmissions, but both transmissions and receptions were performed on the ChipSats. A TI-RTOS program had already been developed by Dr. Hunter Adams [14] for his “Monarch” PCB boards as part of his thesis work. The program included the use of an IMU, a GPS, an analog-to-digital converter (ADC), and radio communication (RF). However, for RF development, only RF tasks were needed. Dr. Hunter Adams’ [15] code was the basis for the new test code. The code had a main script that ran two task scripts: receiving and transmitting. However, the new code needed to change the way the semaphores were posted and pended. The previous code had the semaphores posted and pended in different places in the different tasks depending on the expected execution order. Having only two tasks resulted in a semaphore imbalance. After one transmission, the channel was locked forever. The problem was fixed, and the semaphores were reorganized to append at the beginning of the task and post after finishing the task.

The code worked well for both transmission and reception between each of the two TI launchpads. However, the transmissions were set up to send the raw modulated data without any error correction or filtering. The next section will explain the theory behind the modulation used. The modulation was coded using the EasyLink APIs available in Code Composer Studio. Non-blocking calls were selected so that the packets would be sent without waiting for a confirmation from the receiver. Also, a clear channel checker was added to make sure that the transmitter would only send if the chosen channel was free (not occupied). The following basic sequence is used to send a packet from the launchpad:

EasyLink_setRfPower(14);

EasyLink_setFrequency(915000000);

EasyLink_transmitCcaAsync(&txPacket, lbtDoneCb);

Here, the packet name is txPacket, and the TX done function pointer is lbtDoneCb. The power of the transmission is 14 dBm at 915 MHz. These packets can then be received in the RX tasks with the following API:

EasyLink_receiveAsync(rxDoneCb, 0);

Async was also used on the receiver to make sure the function was also non-blocking. The zero represents the relative start time of the receiver. The APIs used above were able to be programed, with help from the EasyLink API guide [35].

#### RF Signal Modulation with Gaussian Frequency Shift Keying (GFSK)

**Figure 15:**
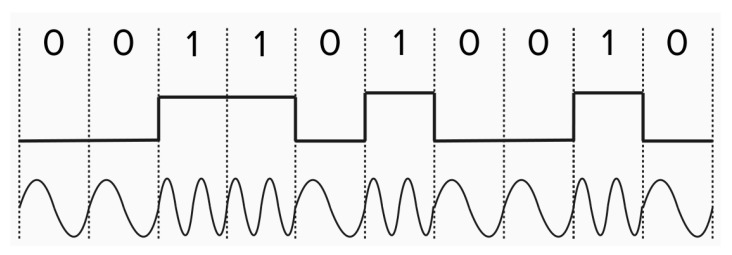
Binary frequency shift keying (BFSK).

The modulation technique used on the ChipSats is frequency shift keying (FSK). The FSK technique was determined by requirement 3.0 (see Table 4). In frequency shift keying, data are sent through a discrete change in the carrier signal frequency, with no effect on the signal power. ChipSats, therefore, use the maximum power available to them to transmit data. The data sent by the ChipSats are coded in binary. With binary frequency shift keying, the carrier signal oscillates between a high and low frequency. The high frequency represents a 1, and the low represents a 0. Figure 15 is an example of an 8-bit carrier signal.

**Figure 16:**
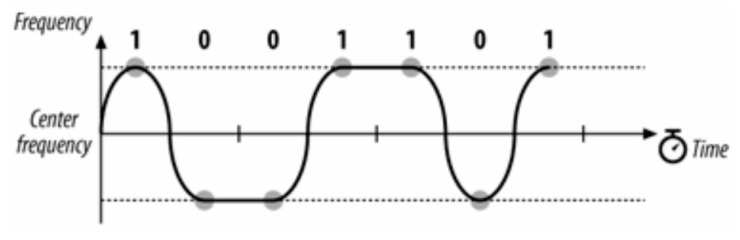
Dual Gaussian frequency shift keying (2DFSK). Note that the *y* axis in this instance displays frequency. Image Credit: “802.11 Wireless Networks: The Definitive Guide” [36].

BFSK is an effective method for the ChipSats because this frequency modulation produces a good signal-to-noise ratio in this instantiation, it has a smaller risk of interference, and the signal power radiates less (it is relatively unidirectional). However, frequency modulation can be expensive. Having to shift frequency (theoretically) instantaneously is straining on the transmitter. It requires costly, powerful, bulky electronic equipment, all of which are unaffordable for a ChipSat. To resolve this issue, the ChipSats use a transmitter that implements Gaussian frequency shift keying (GFSK). The receiver measures the period of change in the incoming signal. In approximately the middle of each of these periods, it reads the frequency of the transmission and translates it into a binary 1 or 0. GFSK allows for gradual frequency changes, lowering the cost of the equipment and decreasing the RF leakage. There are several levels of GFSK. At the base case, a 2GFSK will oscillate between a higher and lower frequency. 2GFSK is what is implemented in the ChipSat modulator. See Figure 16. The transmitter records the frequency changes at each period, as noted below by the gray circles. 2GFSK gives the transmitter the ability to gradually change its frequency.

**Figure 17:**
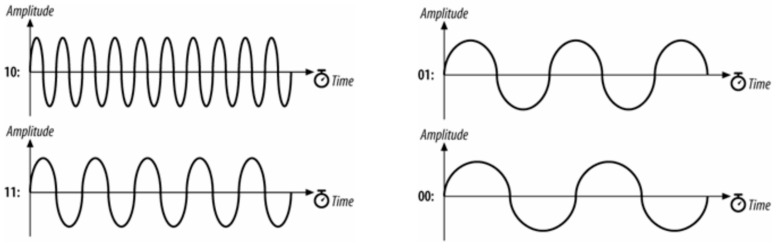
4 GFSKs. Four different frequencies corresponding to the four different possible 2-bit sequences. Image Credit: “802.11 Wireless Networks: The Definitive Guide” [36].

One way to make the transmission faster is to use a 4GFSK. The 4GFSK divides the input signal into sequences of 2 bits. In binary, there are four possible sequences like this: 00, 01, 11, and 10. Each one of these cases has its own carrier frequency. Figure 17 below shows the different separate frequencies.

**Figure 18:**
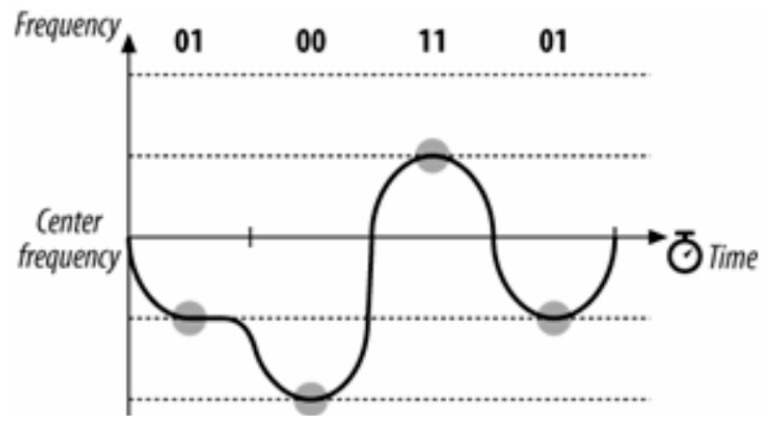
Four-channel Gaussian frequency shift keying (GFSK). Image Credit: “802.11 Wireless Networks: The Definitive Guide” [36].

4GFSK can send and receive data at double the speed of 2GFSK because it can switch between two bits for every frequency change. The next improvement would be to use 8GFSK. Figure 18 below shows an example of an 8-bit carrier signal.

8GFSK is another way to send three bits. But sending three bits only makes the transmission speed 50% faster than 4GFSK. With eight separate frequencies, it is harder, more costly, and more likely to make mistakes because of the many different frequencies to demodulate. For Alpha, neither 4GFSK nor 8GFSK were used on the ChipSats because they made the signal less reliable over long distances. Dual GFSK was used.

### 3.4. Packet Formulation

This section explains how the packets from the ChipSat RF transmitter are formed. The data use forward error correction (FEC) and matched filtering (MF) to increase reliability. These methods were suggested by Dr. Zac Manchester because they are often used in satellite communication. They are not common for small sat communications. But both FEC and MF improve the signal-to-noise ratio, which is needed for long-range transmissions. Matched filtering also helps separate the signal from the noise floor. Since the power budget is low, another way to make the signal more reliable is to add extra bits to the signal. Padding also works like a gain-of-sorts. By repeating the original binary sequence when coding it, if the signal faces interference, the original data can be recovered from the repetition. Also, these techniques make the transmitted data more secure since the sequences used for MF and FEC encoding would have to be known to decode the signal (more detail in the relevant sections).

#### 3.4.1. Raw Data

**Figure 19:**
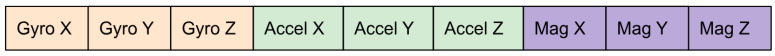
Data packet formulation.

ChipSats transmit data synchronously in packets that include data from each sensor on board. This meets requirement 2.0, which is to periodically record IMU data (see Table 4). Also, packet transmission enables the sample data to have a single timestamp and a complete dataset for each transmission. A benefit of using a single timestamp is that it allows for consistent parsing of the data from each transmission. Since ChipSats do not have unique identifiers for each sensor, knowing the chronological order of the datasets can help recognize each component. The data can be identified based on the expected transmission order. Figure 19 below shows how the packet is made from the IMU values on the ChipSat.

The packets were initially made to only send the IMU data for simplicity. The packets begin by sending three gyroscope values for each direction, and then the accelerometer and magnetometer values (see requirement 2.2 in Table 4). There are nine values that would be transmitted in total. Later, we will add data from the GPS and a temperature sensor. For each of the nine transmitted values, we wanted 16-bit resolution, as per requirement 2.1 (see Table 4). Since the RTOS was written to send the packet in 8-bit pieces, each of the values was divided into two halves. So, 18 8-bit data transmissions are sent in total.

#### 3.4.2. S/N Ratio

One way to improve the signal-to-noise ratio (S/N) ratio when sending signals is to increase the signal’s amplitude with a gain. This needs more power and makes the signal stronger so that the noise around it does not affect it as much. But, because the ChipSat mission has very little power available, other ways had to be used to make up for the S/N ratio. The two ways used were forward error correction (FEC) and matched filtering. These methods add extra bits that repeat the data. If a bit changes from one to zero or zero to one (a bitflip), the original data can be recovered from the repeated data that were sent.

#### 3.4.3. Error Correction

The Alpha mission’s ChipSats employ forward error correction (FEC) techniques (nonlinearly related to SNR) [37] in their transmission to meet requirement 4.0 (see Table 4). FEC is a usual method in radio transmissions that adds extra bits to the signal to enhance robustness. FEC helps reinforce the signal so that it does not need to be retransmitted, and thus makes communication more dependable. For ChipSats, each 8-bit transmission is first encoded using a generator matrix. The generator matrix is a matrix of size 8 by 16 that multiplies the 8-bit signal to generate an encoded 16-bit output. The equation below shows the relationship, where *m* is the original signal, *G* is the generator matrix, and *c* is the resulting 16-bit output.

(1) c=mG

The generator matrix was constructed by joining an 8 by 8 cross-correlation matrix, *P*, with an 8 by 8 identity matrix, *I*. Matrix *G* thus keeps a copy of the original data and a cross-correlated version. Equation (2) shows the equation that appends the generator matrix.

(2) $$G=[P\;|{\;I}_{K}]$$

Equation (3) below shows the generator matrix that Alpha uses. Dr. Zac Manchester created this matrix (Manchester 13).

(3) $$\left[\begin{array}{l} 1\;\;0\;\;0\;\;1\;\;1\;\;1\;\;1\;\;0\;\;1\;\;0\;\;0\;\;0\;\;0\;\;0\;\;0\;\;0\\ 0\;\;1\;\;0\;\;0\;\;1\;\;1\;\;1\;\;0\;\;0\;\;1\;\;0\;\;0\;\;0\;\;0\;\;0\;\;0\\ 1\;\;1\;\;0\;\;0\;\;1\;\;1\;\;0\;\;1\;\;0\;\;0\;\;1\;\;0\;\;0\;\;0\;\;0\;\;0\\ 0\;\;1\;\;1\;\;0\;\;0\;\;1\;\;1\;\;1\;\;0\;\;0\;\;0\;\;1\;\;0\;\;0\;\;0\;\;0\\ 0\;\;0\;\;1\;\;1\;\;0\;\;0\;\;1\;\;1\;\;0\;\;0\;\;0\;\;0\;\;1\;\;0\;\;0\;\;0\\ 1\;\;1\;\;1\;\;1\;\;0\;\;0\;\;1\;\;0\;\;0\;\;0\;\;0\;\;0\;\;0\;\;1\;\;0\;\;0\\ 0\;\;1\;\;1\;\;1\;\;1\;\;0\;\;0\;\;0\;\;0\;\;0\;\;0\;\;0\;\;0\;\;0\;\;1\;\;0\\ 1\;\;1\;\;0\;\;1\;\;0\;\;1\;\;1\;\;1\;\;0\;\;0\;\;0\;\;0\;\;0\;\;0\;\;0\;\;1 \end{array}\right]$$

The data have a hamming distance of five after applying FEC. It can tolerate dive lost receptions or up to two-bit errors without affecting data integrity. Each 8-bit data block from the packet is multiplied by the generator matrix at the start of the TX task. To speed up the computation, only the left half of the generator matrix was used, because the right half just adds the original matrix (although the right half of *G* is an identity). Binary matrix operations were used instead of regular matrix multiplication for coding FEC into the flight code because the data is in binary.

**Figure 20:**
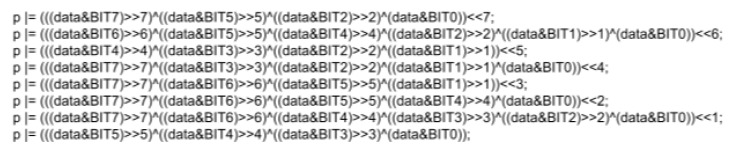
Binary matrix multiplication of the LHS *G* matrix with the data.

Binary matrix multiplication follows the same procedure as regular matrix multiplication. Each of the values in the columns of *G* is multiplied by the corresponding value in the data vector, and the values are summed to produce a scalar value in the output vector. Binary multiplication uses the “binary and” operator, which is “&” in *C*. It only produces a binary 1 when both factors are 1, so only the entries in *G* with a 1 are relevant. For the first column, those are entries 7, 5, 2, and 0. Like matrix multiplication, the resulting values are added to produce a scalar value. The values are first shifted right to the same position, using the double greater-than symbol in *C*, followed by the number of shifts (e.g., “>>4” means right four positions). The values are put in the zero-index position, so they are in the same column. Then, a “binary or” is used to sum them. In *C*, the “binary or” operator is the “^”. Finally, the value is shifted left to move it from the zero position back to the original position. The left-bit shift also uses two carrots but points left (double less-than symbol). See Figure 20 below for the binary multiplication code.

**Figure 21:**
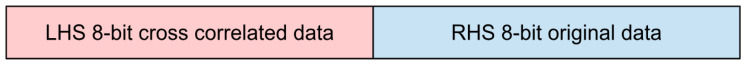
Appended FEC.

The left half of the FEC-encoded data is obtained by performing these operations. The code used to calculate the “*p*” vector is shown in Figure 20. The right half of the FEC encode is the same as the original data, so the left half and right half are joined to make the final 16-bit vector. See Figure 21.

After adding the data, the FEC-encoded message can also add preamble identifiers. The message length increased by two times, so the signal-to-noise ratio also went up by two with FEC. The packet length now is two times bigger, from 18 8-bit sequences to 36 8-bit sequences.

#### 3.4.4. Pre- and Post-Amble

**Figure 22:**
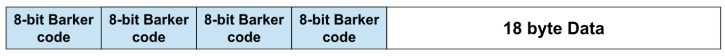
Addition of a preamble.

To help with demodulating the signal, the data can have identifiers added to the packet after FEC. The packet usually has an identifier at the start and the end to distinguish where the packet begins and ends. A preamble is an identifier that comes before the packet, and a post-amble is one that comes after the packet. For the Alpha mission, only a preamble was used. This fulfilled requirement 5.0 (see Table 4). The preamble helps the demodulator identify the signal and find where each packet starts. This way, the signal can be captured and demodulated. For Alpha, the preamble consisted of four 8-bit Barker codes (following requirement 5.1 in Table 4). After the Barker codes, the FEC-encoded data came. See Figure 22 below.

If the transmitter and receiver agree on the same Barker codes, they can use any value they want. These four 8-bit Barker codes make sure that the data sequence is distinct and recognizable. In other words, a preamble of this size prevents the signal from being confused with a random noise. The packet length became 40 8-bit transmissions by adding 4 8-bit identifiers.

#### 3.4.5. Filtering

**Figure 23:**
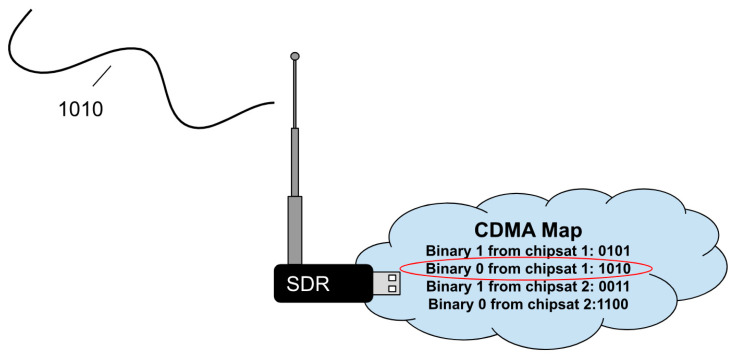
CDMA allows the receiver to recognize which device is transmitting.

The ChipSats used matched filtering as the filtering technique, as required by requirement 6.0 (see Table 4). Matched filtering not only boosts the S/N ratio and adds signal rigidity like FEC, but it also allows for greater theoretical gain. It does this using a 64-byte sequence for each binary bit, which are called PRN codes. Specifically, the codes used for alpha are gold codes. Each ChipSat has two PRN codes: one for sending a binary 1 and the other for sending a binary 0. Matched filtering also has the unique feature of enabling multiple devices to use the same communication channel. This is known as code division multiple access (CDMA), and it works by assigning different pairs of PRN codes that are as orthogonal as possible to each device. The receiver then adjusts to detect the set of PRN codes that are being sent. See Figure 23 for a graphical explanation.

The number of devices that are using the CDMA determines how the PRN codes are selected. The PRN codes are created to maximize the orthogonality between codes. Orthogonality is essential to optimize the allowable hamming distance. For example, with only one device, one could choose to send a binary zero 511 times and a binary one 511 times. But with multiple devices, to distinguish which device is transmitting the data, the PRN codes cannot be exactly opposite. So, the more devices in a system, the more likely there is cross-correlation. Fortunately, in Alpha, only two ChipSats are used, so there are four PRN sequences. With only four PRNs, a hamming distance of 127 or 63 possible bit flips is achieved. In the ChipSat flight code, bits for future matched filtering are added after FEC. The respective PRN sequences are defined at the start of the TX task. A set of “for” loops runs through the preambles and each of the FEC-encoded bits. An “if” statement assigns them either PRN [0] or PRN [1] based on the respective binary value. The final transmitted parcel is built in order by appending each of the 16 PRN sequences (from the 16-bit FEC-encoded data). The final transmitted parcel length is 1024 bytes. Since there are 22 parcels, that makes each packet length a total of 22,528 bytes.

### 3.5. Ground Station

To complete a successful RF transmission as required by 7.0 (see Table 4), a ground station had to be set up to receive the signal. Two ways of setting up a ground station were attempted, and both will be used for Alpha. The first way was to use software-defined radio (SDR). This would let Alpha team members and other enthusiasts try to pick up the signal. The second way was to use a receiver network. This could allow a larger network to track the signal and report back any data gathered.

#### 3.5.1. Software-Defined Radio RTL-SDR

An SDR was the device we used for the Alpha ground station. We picked the SDR because it can do many different things with radio signals. Usually, radio receivers have hardware parts that are made for a certain kind of signal. With an SDR, the software does all the work on the signal. Things like mixers, filters, amplifiers, modulators/demodulators, and detectors are all performed by the software, not the hardware. For Alpha, we used a Realtek RTL2832U SDR (RTL-SDR) [38,39]. The RTL-SDR is a cheap SDR that uses DVB-T TV tuners to adjust the signal and has an RTL2832U chip. It talks to a COMs channel through a USB port.

#### 3.5.2. Verifying Transmitting Using SDR Sharp

**Figure 24:**
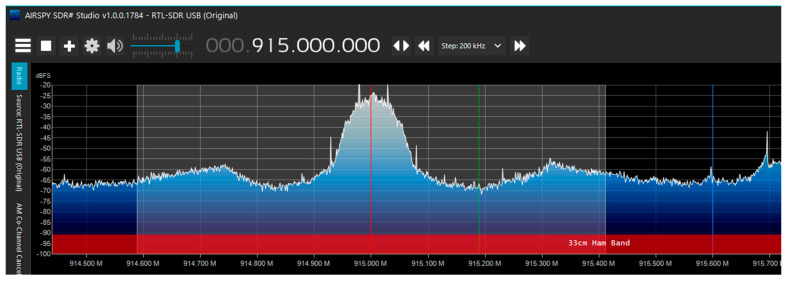
AIRSPY dashboard identifies ChipSat transmissions at 915 MHz.

To confirm that the RTL-SDR worked, it was tested to see if it could detect the transmissions sent by the TI launchpad. SDRSharp, which is SDR software v1.0.0.1784 developed by AIRSPY, was used for the test. AIRSPY connects to the RTL-SDR through the COM port and records signals within a specified range. SDRSharp can be downloaded from the AIRSPY website and configured with the RTL-SDR [38,39]. After setting up SDRSharp with the RTL-SDR, the TI launchpad can be plugged in. The RTL should be able to receive the packages if they are transmitting. The packages can be seen as a visual representation at the top of the screen. Figure 24 shows how the AIRSPY dashboard looks when it receives the ChipSat transmissions at 915 MHz.

#### 3.5.3. Receive FEC Signal on Raspberry Pi

The final step to finish the SDR ground station was to receive a signal sent from the TI launchpad using the RTL-SDR and conduct both the matched filtering and the forward error correction. A script that could demodulate an unencoded raw signal had already been written by Dr. Hunter Adams [15]. The script was made to demodulate a signal with no encoding. The script by Dr. Hunter Adams was meant to run on a Raspberry Pi connected to the RTL-SDR. The script would look for the four 8-bit Barker codes before demodulating. The script was run on the Raspberry Pi 4 Model B. Unencoded GFSK data were successfully received by the Pi, demodulated, and saved in a .txt file. This satisfies requirement 7.1 (see Table 4). Additionally, FEC was added to the demodulating code. Every two 8-bit transmissions were repeated again to recreate the 16-bit FEC vector. The 16-bit vector was then multiplied by the parity check matrix, *H*, to obtain the original data back. The parity check matrix can be made by adding the identity matrix, *I*, to the negative transposed cross-correlation matrix, *P*. See Equation (4) below:

(4) $$H=[I_{K}\;|-P^{T}]$$

The parity check matrix will mathematically fix errors within the allowable hamming distance of the received transmission. The code for demodulating included the parity check demodulator. A transmission with FEC encoding was sent from the TI launchpads to test the code. The signal was decoded correctly and successfully. The following step was to figure out how the ground station script could achieve matched filtering. Reversing matched filtering was hard because the search algorithm in the demodulating script could not recognize the preamble anymore since each Barker code was filtered. The answer to the problem is not covered in this manuscript. However, a different method for demodulation was discovered, and complete demodulation of a FEC and match-filtered signal can be performed.

#### 3.5.4. Tiny GS Satellite Balloon Launch

**Figure 25:**
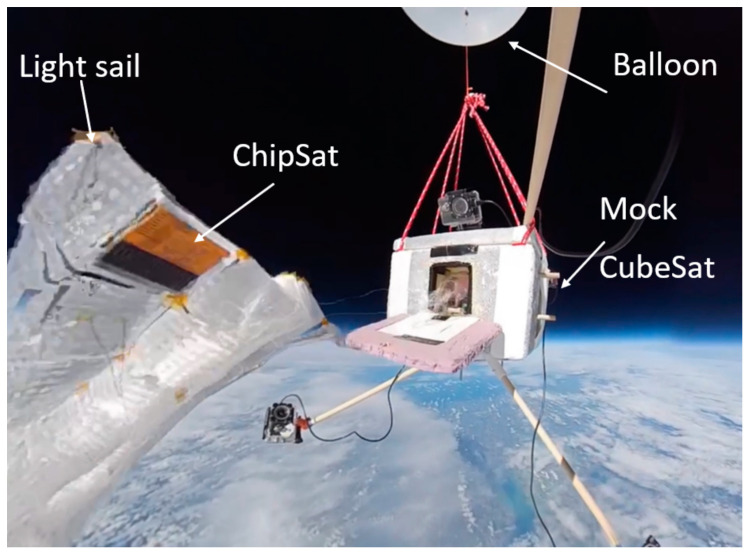
Photo from a balloon launch.

If the ChipSat transmission could not be detected by our SDR, another alternative was to use a receiver network. The receiver network, however, depends on the microchip having LoRa (long-range capability). The CC1310 does not have this feature, but the next-generation ChipSat will be improved to include it. The receiver network was tried several times, one of them during a balloon launch on 10 October 2021. The aim was to attempt and set up a connection with the LoRa on the ChipSat during the mission using an Adafruit feather receiver. The balloon launch was performed to test the operation of the satellite and to capture footage of our satellite to raise awareness of our mission. See Figure 25 below for a picture taken by the satellite from the balloon.

To communicate with the LoRa transceiver, a TinyGS ground station (consisting of an Adafruit feather and antennae) was selected. The TinyGS system enables anyone around the world to receive signals from LoRa satellites or any other flying device that uses low-power communication techniques. The TinyGS module uses an ESP32 board. It can communicate with the sx126x and sx127x LoRa transceivers. TinyGS has online instructions to help with the setup. In brief, the TinyGS software (version 21021701) is installed to connect to the TinyGS system. After the setup is completed, the TinyGS board can be plugged into the computer.

**Figure 26:**
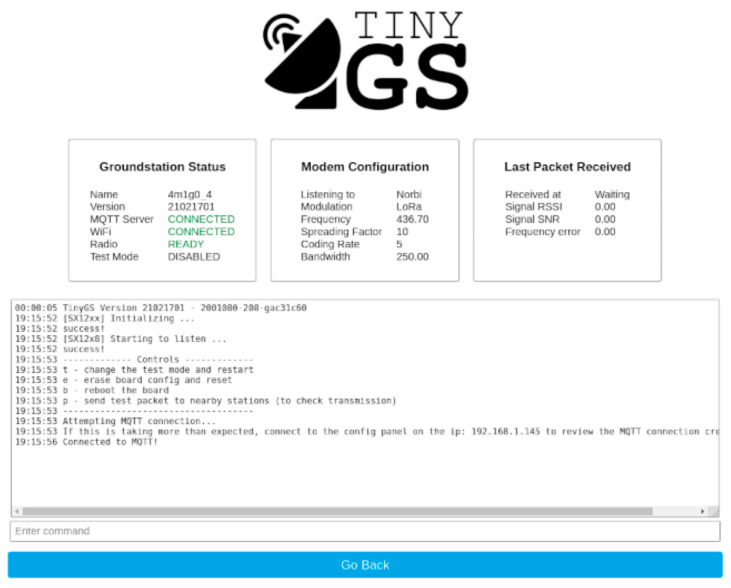
Example TinyGS dashboard.

The software will recognize it, and an IP address will show up on the LCD screen on the board. At this point, the operator will connect their laptop WiFi to the TinyGS SSID. Once connected, the IP address shown on the LCD screen can be entered in the web browser. The connected device will show a dashboard that will allow the user to set the parameters of the ground station. After that, the user can listen to the ChipSat LoRa through the TinyGS system. The received packets will show on the dashboard of the network. Figure 26 below shows an example of the dashboard.

**Figure 27:**
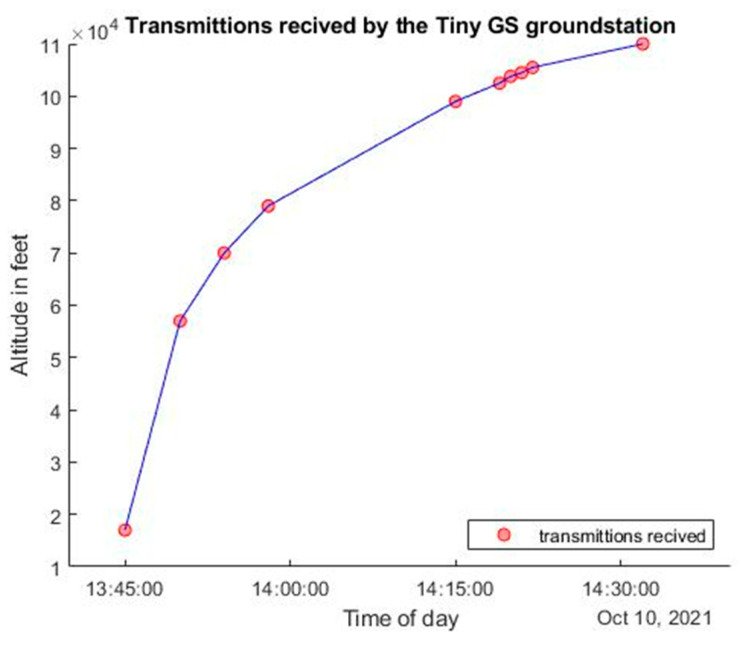
Satellite height as a function of TinyGS transmission timestamps.

When the CubeSat balloon launch happened, packets were not yet ready to be sent. However, the test was performed to prove that we could successfully receive signals from the ChipSat LoRa transmitter. Although the transmissions could not be decoded, a timestamp was shown when the TinyGS ground station connected to the ChipSat (inside of the balloon payload). Each time the signal was detected, the timestamp and current height of the satellite were recorded. The current height was measured using GPS onboard the balloon payload. Figure 27 below shows the time of the transmissions we received and the respective height of the CubeSat.

The experiment showed that the CubeSat could still send a signal when it was close to 100 k ft. In summary, a basic validation of the TinyGS system as a possible receiver for Alpha was performed.

## 4. Attitude Control System (ACS) Development

One of the key functions of a satellite is to manage angular momentum. The subsystem that handles this function is called the attitude control system (ACS). For Alpha, the ACS system must be able to stop tumbling, stabilize spinning, and orient the satellite. See Table 5 below.

### 4.1. Duties of the CubeSat ACS

**Figure 28:**
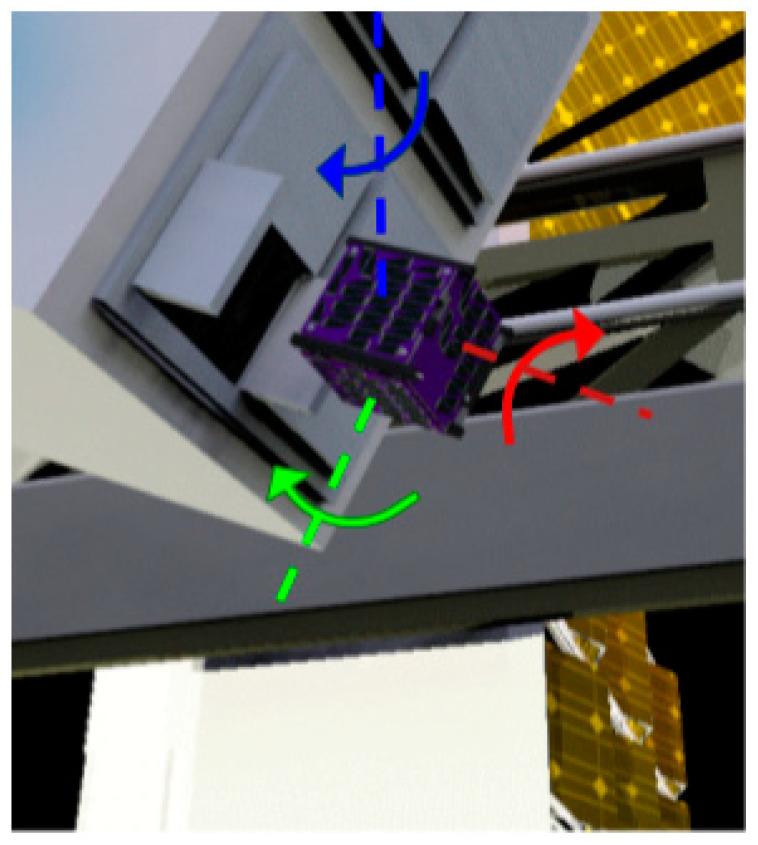
CubeSat detumble after deployer ejection. The blue, red, and green arrows symbolizes the initial spin of the satellite along its three respective axes. Image Credit: Josh Umansky-Castro.

The CubeSat deployer does not provide stable ejection when it releases the CubeSat from the International Space Station (ISS). The CubeSat’s initial spin is unknown. However, the CubeSat deployer ensures that the CubeSat’s angular speed will not exceed five degrees per second. It is crucial to reduce any remaining momentum, as it would make connecting with the CubeSat difficult. Requirement 1.0 in Table 5 specifies that the leftover momentum shall be reduced by 10% of the leftover angular speed in the x and y directions. Figure 28 illustrates the CubeSat’s ejection.

**Figure 29:**
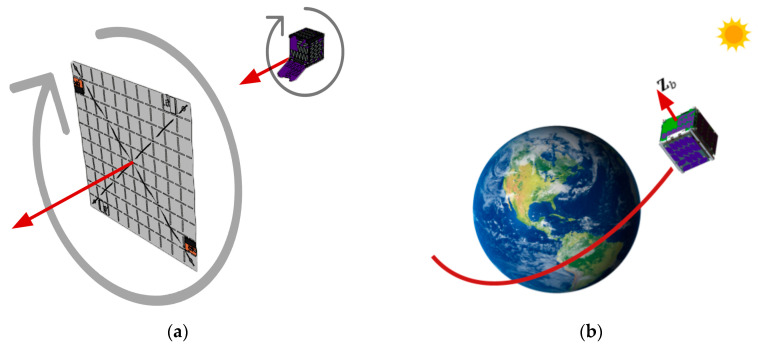
(**a**) CubeSat and light sail spin stabilization; (**b**) CubeSat achieves pointing. Image Credit: Josh Umansky-Castro.

At the same time, the CubeSat is made stable by spinning. When the CubeSat rotates around its largest axis of inertia, it can resist any kind of disturbance. Also, it ensures that the light sail will have the same stability after it comes out. A common example of spin stabilization is how a frisbee keeps its balance by spinning when it is thrown. The frisbee can stay stable around its largest axis even if there is wind or other interference. Requirement 2.0 states that the final spin should be 1 rad/s around the *z* axis, with a margin of 10%. Having this spin condition for both the CubeSat and the light sail will ensure stability. As a result, stability improves the reliability of the communication between the transceivers on the ground station and the satellite. Another advantage is that the steady rotation will provide active cooling for the CubeSat. Spin helps prevent any one side from being exposed to the sun for too long. Figure 29 shows how spin stabilization will be achieved for both the CubeSat and the light sail. 

The last essential part of the ACS is to orient the satellite. Table 5’s requirement 3.0 aims to make it more likely that the *z* axis will point to space. A space-pointing *z*-axis ensures good radio signal strength to the ground and would also assist with the light sail deployment. Both the positive and negative Z sides of the CubeSat must be kept at right angles to Earth’s surface. Figure 29 above shows the desired pointing of the CubeSat.

### 4.2. CubeSat ACS Actuators

The most common ACS systems for modern satellites use ion propulsion, reaction wheels, or thrusters. Even though satellites may have magnetic torque coils, they also choose another propulsion method with a higher specific impulse. Our CubeSat is different because it only uses magnetic torque coils. But, since our CubeSat is small and light and has stabilization requirements, magnetic torque coils could be enough. The benefit of using magnetic torque coils is that they are small, cheap, very easy to control, and relatively low in energy.

**Figure 30:**
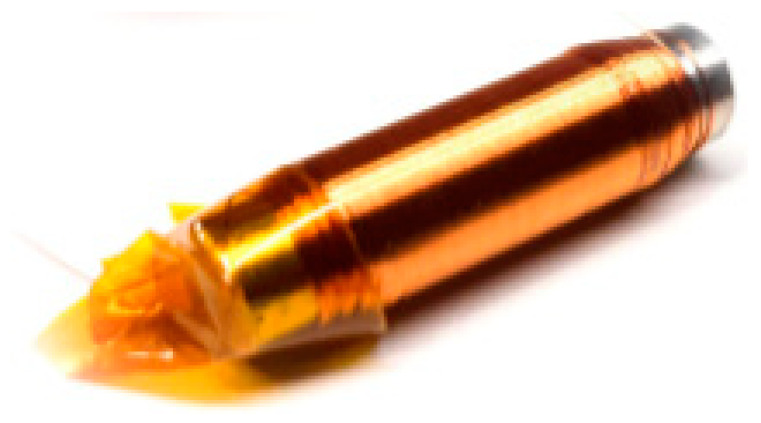
Torque coils made in-house.

Magnetic torque coils are basically electromagnets. We made the torque coils for the CubeSat ourselves. A thin copper wire is coiled five hundred times around a mu metal ferromagnetic core. The core is an inch long and three millimeters wide. It works as a booster. When the electromagnet is on, the polarized dipoles of the ferromagnetic electrons line up. Unlike a permanent magnet, because the core is made from a softer metal, it does not keep its magnetization and almost quickly loses its alignment after the coil is off. The core adds a kind of gain to the magnetic torque coil. The magnetic field strength becomes about 15 times bigger with the mu metal ferromagnetic core. Please see Figure 30 below:

The ACS controller decides how much the torque coils are magnetized. The controller works on the Teensy 3.5 flight computer. The flight computer uses an IMU to measure the actual rotational velocity. Based on the rotational velocity input, the controller gives a pulse width modulation (PWM) value to the coil. Since the flight computer has low power, an H bridge is required. The H bridge powers the torque coils in proportion to the PWM value from the flight computer. The PWM level controls the strength of the magnetic torque coils.

Besides the coils themselves, all the other components are cheap, commercial off-the-shelf (COTS) parts that can be easily bought online. They are not space-rated but rather hobbyist microcontrollers, electronics, and sensors. However, similar products have been used in space with very dependable results. The system, including the flight computer, can be built for less than USD 100. Compared to the cost of other ACS systems on comparable missions, our system was very cost-effective.

### 4.3. Attitude Control

The design of the CubeSat ACS system involved both passive and active methods. The satellite’s shape and the controller gains were adjusted to try to reduce power use. The tradeoffs were between satellite stability and controller robustness. These three factors were fine-tuned so that the ACS system satisfied the system-level requirements.

#### 4.3.1. Passive Techniques

**Figure 31:**
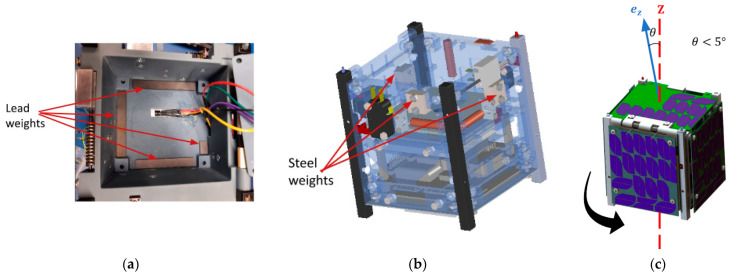
Passive ACS techniques used in Alpha: (**a**) lead weights to increase the moment rotational moment of inertia; (**b**) steel weights to balance the center of mass toward the middle of the CubeSat; (**c**) the rotational *z* axis and the geometric *z* axis within 5 deg for stability. Image Credit: Josh Umansky-Castro.

The CubeSat had limited power, so it used passive methods to control its attitude. The CubeSat’s passive ACS worked by adjusting the mass distribution. Mass distribution had two benefits: it kept the CubeSat balanced on each axis to prevent tilting or shaking, and it made the *z* axis the most dominant principal axis (requirement 2.0 in Table 5). Lead weights were arranged in a square shape in the XY plane. They were in the light sail compartment opposite the electronics. The lead was placed there to make the CubeSat weight even along the *z* axis. Steel weights were also added, pointing along the *z* axis direction. They went around the edge of the CubeSat, up and down the inside of each of the side-facing panels. Having both the lead and steel weights on the outside of the CubeSat made the principal moment of the inertia axis line up with the geometric *z* axis. Figure 31 shows how the mass was placed.

#### 4.3.2. Active Techniques

The CubeSat has a controller algorithm that is an active way to improve the ACS system. The controller algorithm uses two controllers for different purposes: one for spin stabilization and one for axis alignment. This controller was initially developed by Armin [18].

##### Kane Damper

**Figure 32:**
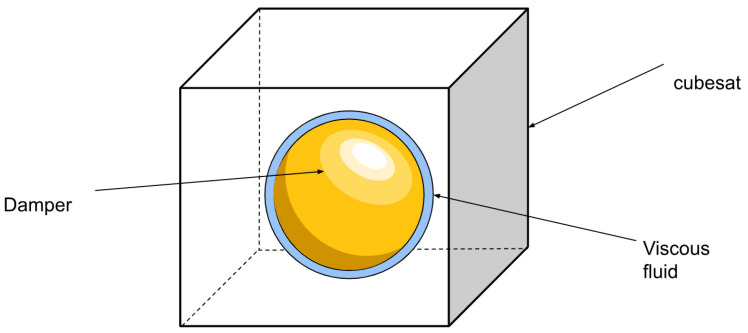
Kane damper model.

The ACS system uses a Kane damper as its first controller. The Kane damper is a controller that imitates a rigid body with a frictional damper acting on it. The algorithm simulates a situation where the spacecraft has a spherical inner chamber. There is a sphere-shaped mass inside this chamber that is slightly smaller, and a thick liquid around it. The CubeSat is slowed down by the imaginary difference in angular speeds between the spacecraft and damper. Figure 32 explains this better.

The simulated situation has two adjustable coefficients: the moment of inertia of the inner sphere, Id, and the damping constant of the viscous fluid, c. The inner mass is simulated as a sphere, so the moment of inertia can be simplified as a coefficient times the identity matrix. Changing the Kane damper can therefore be performed by changing two scalar values. A surface plot can be made, performed by Davide, to pick a set of values that best improve the stability and convergence of the controller (Carabellese) [19].

##### PD Controller

Pointing is the second component of the ACS system. It is activated by a toggle switch in Simulink. It uses a proportional derivative (PD) controller to achieve pointing. A PD controller is a feedback controller that changes its system outputs based on the error, which is the difference between the desired value and the real time value. The PD controller does this by (1) a proportional relation to the error and (2) a derivative relation to the error history. Figure 32 below illustrates the feedback process.

**Figure 33:**
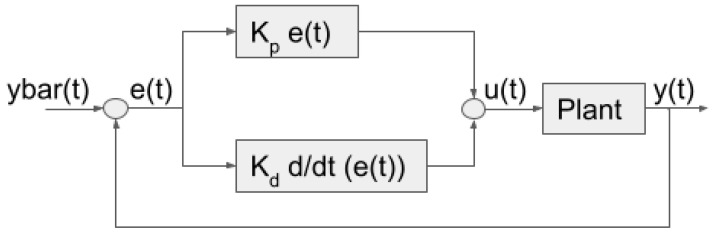
PD feedback block model.

The proportional control is calculated with each feedback loop iteration. The reference value is marked as “ybar” in Figure 33 below. The current value is marked by “y”. The error, “e”, is the difference between the reference and feedback values. The proportional control is adjusted by the gain constant “Kp”. The output can be proportionally scaled to the size of the error.

The derivative control is based on the slope of the line that touches the last two or more feedback loop iterations. The line’s direction is considered to avoid going over the reference value. It can also help counter some disturbances. The derivative control is changed by the gain constant “Kd”. Proportional Integral Derivative (PID) controllers are common and widely used in many industries. Adding the integral component helps counter more disturbances and makes sure there is no steady-state error. However, it also makes the controller more expensive and slower to run. The communication requirement allows for a small steady-state error that the PD controller may have. The requirement for our settling time is to reach a steady state in 8 h. Simulations suggested that settling time would not be a problem. Therefore, making sure of the system’s robustness was more important. This meant keeping the computational load on our flight computer low to reduce risk. Since the flight computer controls the whole satellite, it was decided to keep computation low to lower risk.

##### Validating PD Control Method

We wanted to make sure that the PD controller was the best controller for meeting our requirements, so we compared our system to the common controllers in the industry. The comparison included the P + V controller, the P + V Double Integrator Controller, the Double Integrator Gain Tuning Controller, the 2DOF Feed Forward Controller, the P + V Control Law Inversion Controller, the Open Loop Guidance Controller, and the Real-Time Optimal Controller. These are the standard controllers in the field. For the final control report, we tested and compared six other controllers based on controller quadratic cost, computation time, rise time, and stiffness. We measured these values based on how well the controller could move from rest to a position of one without any units. We then compared the results to those of a PD controller. Table 6 below shows the results.

The different controllers have trade-offs, so to establish optimality the results depend on the specific system and its needs. For the CubeSat, low quadratic cost was the optimization criterion. Quadratic cost is a measure of acceleration over a time interval, which relates to power usage. Requirement 4.0 in Table 5 states that the power must be below 0.9 watts. This requirement was the most restrictive for the control system since the rise time requirement (requirement 3.0 was easily satisfied by all the controllers). As shown in Table 6, the PD controller has the lowest quadratic cost. To further verify the robustness of the PD controller, the rise time was measured as a function of the timestep. A low sensitivity would fulfill requirement 5.0. Since the Teensy microcontroller runs both the ACS and the flight code, the sensitivity of the rise time was tested to reduce the computational load for the ACS and integrate the controller with the rest of the flight software. It was found that the timestep had very little impact on the rise time. The flight computer could therefore run at a larger timestep if needed. See Table 7 below.

The PD controller was confirmed as the best choice for our ACS system because of the minimal quadratic cost and the stability of the rise time for different timesteps.

#### 4.3.3. The Controller Software

**Figure 34:**
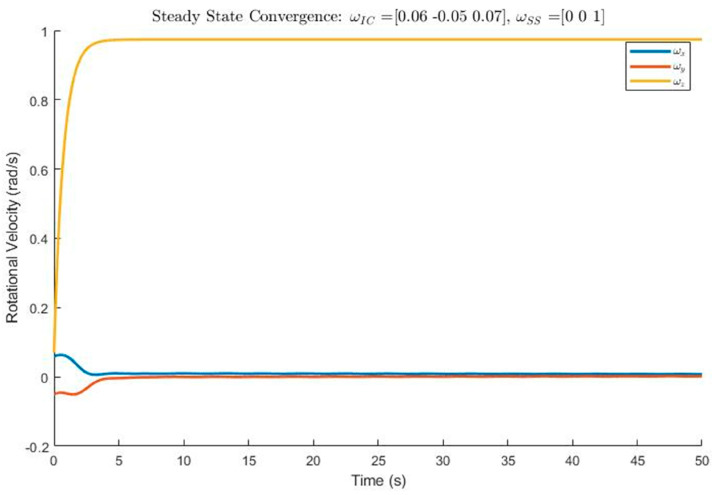
Unrealistic convergence of Kane controller.

The ACS software for Alpha is built in Simulink with the parameters set in a MATLAB^®^ script. The Simulink model has a controller that can switch between the Kane damper and the PD controller when needed. A plant was added to simulate the system dynamics. The final goal was to put the controller that was tuned in the end into the Arduino flight code as a C++ library file. Davide Carabellese created the original code for his master’s thesis [19]. However, traceability was needed to meet the system requirements for the CubeSat ACS. Traceability began with naming each flow with the correct variable. Also, at every point where something interacted, the equation that was used was referenced. Referencing each calculation made it possible for outside experts to easily check the control algorithm. Each step could be linked back to a basic equation, ensuring the algorithm was accurate. After traceability was performed, the controller was checked for functionality. A Verification Cross Reference Matrix (VCRM) was used to check for functionality. The VCRM showed the changes that were needed in the code. Some of the values that were changed were the starting spin conditions, expected orbital inclination, orbital height, the mass of the CubeSat, the number of loops in each coil of the magnetorquer, and the area of the magnetic torquer. These variables were redefined in the MATLAB^®^ script. The controller was checked by running a simulation with a plant model of the dynamics. When the simulation ran, it was clear that a certain parameter made the controller achieve stable steady-state values too quickly. See Figure 34 below for the first results of the simulation.

The CubeSat ACS parameters were tested for validation after seeing the simulation outcomes. The first try was to revise the principal moment of inertias. Experimental moments of inertia values were needed because the values estimated by SOLIDWORKS lacked many of the smaller components, making their evaluation artificially low.

##### Experimentally Calculating the Moment of Inertia of the ChipSat

The angular acceleration is found using Newton’s second law of rotation and acquiring Alpha from the net torque that the magnetic torque coils apply and the CubeSat’s moment of inertia. So, to make the CubeSat stable dynamically, the moment of inertia needs to be known well. See Equation (5) below.

(5) $$\begin{array}{c} I_{x}{\dot{\omega }}_{x}+\left(I_{y}-I_{z}\right)\omega_{y}\omega_{z}=M_{x}\\ I_{y}{\dot{\omega }}_{y}+\left(I_{x}-I_{z}\right)\omega_{x}\omega_{z}=M_{y}\\ I_{z}{\dot{\omega }}_{z}+\left(I_{x}-I_{y}\right)\omega_{x}\omega_{y}=M_{z} \end{array}$$

**Figure 35:**
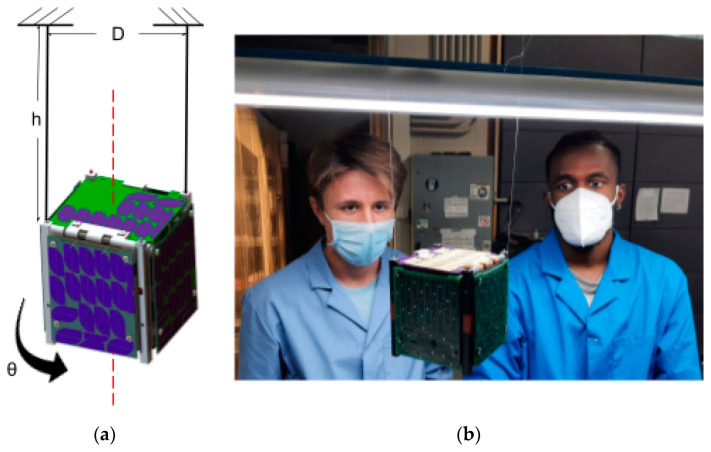
Moment of inertia testing setup [40]. (**a**) Diagram of setup; (**b**) Live picture of setup as tested.

The SOLIDWORKS model was used to initially estimate the moment of inertia, but it did not account for many of the wires and other components that were hard to draw. To check the values obtained and assess their precision, a more accurate calculation was required. The moment of inertia was then calculated experimentally by measuring the pendulum motion of the CubeSat spinning around its axes. The experiment involved setting up a bifilar pendulum. The pendulum was made by hanging the CubeSat from two fishing lines at a height, h, from the hang point to the pivot point. The two fishing lines were spaced diagonally across the CubeSat at a distance, *D*. Distance *D* was kept constant (the fishing lines were vertical). Figure 35 below shows the experiment setup. 

The setup in Figure 35 was used to give the CubeSat a small initial rotational displacement. The oscillations’ period was computed. To reduce human error, the period of three successive oscillations was measured and then corrected (divided by 3). For more accuracy, the harmonic evaluation was repeated five times and averaged again. A final period was obtained. The CubeSat was spun around the other two axes, and the experiment was performed again. The periods were calculated around the other two axes. Equation (6) below shows the equation for the period of a bifilar pendulum. Note that Equation (6) is based on a small displacement angle.

(6) $$T=\frac{4\pi }{D}\cdot \sqrt{\frac{hI}{mg}}$$

Deriving for the moment of inertia, Equation (7) is solved for the following:

(7) $$I=\frac{mgD^{2}T^{2}}{16\pi^{2}h}$$

Using the known values of the CubeSat’s mass, the separation distance, and the bifilar pendulum’s length, the periods recorded allowed for the calculation of the moment of inertia in each of the principal directions. The inertia matrix was determined using the principal moments of inertia that were found. Since the alignment of the maximum principal axis of inertia was within 5 deg of the *z* axis, the off-diagonal elements were negligible and made zero. Equation (8) below shows the resulting inertia matrix. The units of the inertias are grams per squared millimeter.

(8) $$\left[\begin{array}{l} 2,109,759.45\;\;\;\;\;\;\;\;\;\;\;\;\;\;0\;\;\;\;\;\;\;\;\;\;\;\;\;\;\;\;\;\;\;\;\;\;\;0\\ \;\;\;\;\;\;\;\;\;0\;\;\;\;\;\;\;\;\;\;\;\;\;\;1,983,906.17\;\;\;\;\;\;\;\;\;\;\;\;\;\;0\\ \;\;\;\;\;\;\;\;\;0\;\;\;\;\;\;\;\;\;\;\;\;\;\;\;\;\;\;\;\;\;\;\;0\;\;\;\;\;\;\;\;\;\;\;\;\;\;2,308,281.50 \end{array}\right]$$

The CAD estimated values are shown below. Refer to the values in Equation (9).

(9) $$\left[\begin{array}{l} 2,050,068.12\;\;\;\;\;\;\;\;\;-1682.74\;\;\;\;\;\;\;\;\;\;\;\;\;\;-1901.26\\ \;\;-1682.74\;\;\;\;\;\;\;\;\;\;\;1,652,976.53\;\;\;\;\;\;\;\;\;\;\;81,119.53\\ \;\;-1901.26\;\;\;\;\;\;\;\;\;\;\;\;\;\;81,119.53\;\;\;\;\;\;\;\;\;\;\;2,193,395.14 \end{array}\right]$$

**Figure 36:**
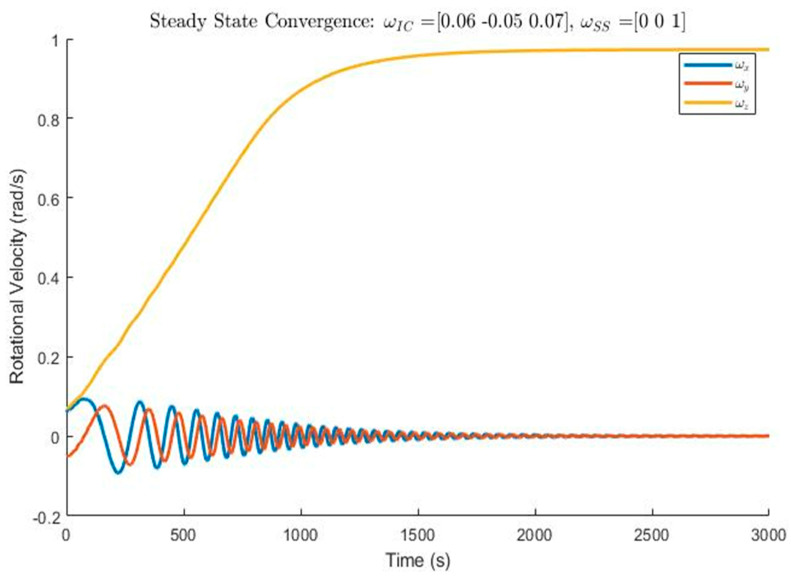
True convergence of Kane controller.

The calculated values are very close to the SOLIDWORKS predictions. The principal axis moments are the main factors that affect the CubeSat’s behavior and have similar sizes. All the off-diagonal entries are much smaller in size by a factor of 20–100 times. Therefore, it can be reasonable to ignore them. The calculated principal moment values are all higher in proportion than the SOLIDWORKS ones. The difference is explained by the wires and other features that the SOLIDWORKS model does not include. The values derived from the experiment were always larger. Although the experimental values were 5–20% bigger, the difference was not big enough to be the only reason for the fast rise time shown in Figure 36. Therefore, the next verification was performed to check the magnification factor of the mu metal rod in the magnetic torque coils.

##### Calculating the Amplification Factor of the Mu-Metal Core

To measure the magnetic strength of the torque coils precisely, we needed to know how much the mu metal rod amplified it. We had used an estimated amplification factor before based on values from material charts, but we had never tested it experimentally for our system. To achieve the amplification factor of the torque coils on the CubeSat, we had to find the magnetic dipole. We used the IMU magnetometer to measure the dipole. Dr. Lee derived the equation we used to calculate the magnetic dipole of the coil [41]. See Equation (10).

(10) $$M=\frac{4\pi }{\mu_{0}}\;\cdot {\left[\frac{\frac{R_{x}}{L}-\frac{1}{2}}{{\left({R_{x}}^{2}-R_{x}L+\frac{L^{2}}{4}\right)}^{3/2}}-\frac{\frac{R_{x}}{L}+\frac{1}{2}}{{\left({R_{x}}^{2}-R_{x}L+\frac{L^{2}}{4}\right)}^{3/2}}\right]}^{-1}$$

*R_x_* is the distance between the coil and the magnetometer, and *L* is the coil’s length. The units for the magnetic field, *B*, are Teslas. By knowing the magnetic dipole of the magnetorquer, the amplification factor could be calculated from the current that was applied (*I*), the coils that were used (*N*), and the area of the cross-section (*A*). Refer to Equation (11):

(11) $$AF=\frac{M_{dipole}}{NIA}$$

An Arduino program was created to switch the magnet coils on and off. The coil’s radius and length were measured. The coil’s distance from the IMU was also recorded. The magnetic field was found to be 55 μT. This was derived to be a magnetic dipole of 0.0619 amps·m^2^. The amplification factor was calculated using Equation (11) to be ~13.5 to 14. The experimental result for the amplification factor was much lower than the theoretical one. The amplification factor was about 10% of what was expected before.

##### ACS Verification Conclusion

The controller code was checked and confirmed, and the results are shown below. The x, y, and z axes were given initial tumble angular speeds of 0.06, −0.05, and 0.07, respectively. Refer to Figure 36.

Euler’s coupled equations of motion were used to mathematically verify the model. The rotational motion along the *x* and *y* axes is not relevant for the *z* axis rotational motion since they are both zero. The simplified equation yields an asymptotic exponential increase function. The velocities along the *x* and *y* axes, on the other hand, are very interrelated. The general solution to these coupled equations shows sinusoidal behavior. This matches the prediction, as seen in Figure 36. The convergence time is more acceptable. The controller is expected to take 25–35 min to stabilize the CubeSat. The oscillations can also be theoretically explained by the damping of the system. Looking at Figure 36, the system appears to be nearly critically damped. A critically damped solution satisfies requirements 1.0 and 2.0 since the system is successfully detumbled and spin stabilized. The TRL of the control system increased from a three to a five due to the measures discussed above. The improvements in TRL were achieved through adding traceability of the model to the respective requirements, verifying the constants, and conducting a rigorous validation of the parameters. Further technological development could be performed by testing the controller experimentally in an air-bearing environment and ultimately launching a CubeSat into low-earth orbit (LEO) to test the rotational kinematics.

#### 4.3.4. The Hardware

After the software simulation stage was completed by the ACS code, the hardware ACS could be tested. Software was first used to confirm that the ACS actuation matched the controller’s outputs for different scenarios and to check that the plant model behaved in reality as expected by theory.

#### 4.3.5. Tested Software on Teensy Flight Computer

All the hardware components in the ACS system were checked by verification testing. The microcontroller was the first component to be tested. It is a Teensy 3.5 microcontroller, and it is used for Alpha. The CubeSat’s flight software is run by the Teensy 3.5 controller. The testing of the microcontroller’s hardware was to make sure it met requirement 5.0 in Table 5.

The ACS controller algorithm was added as a library [13] to the flight software of the Teensy and then tested. The script was run using the plant model in the code of the Teensy. Controller runtime, computational speed, and accuracy were measured and plotted. These plots were compared with the MATLAB^®^/Simulink simulations. No major differences were seen. Different timesteps were tested. A refresh rate of 0.01 s was chosen for using the Teensy. This timestep is a safe estimate because the Teensy was also running the plant simulation, which it will not do when it is deployed. A test was performed where the Teensy would keep running until the plant had steady values. The test was repeated three times consecutively. The Teensy was verified to be able to run the ACS control algorithm.

#### 4.3.6. Magnetic Torque Coil Endurance Tests

**Figure 37:**
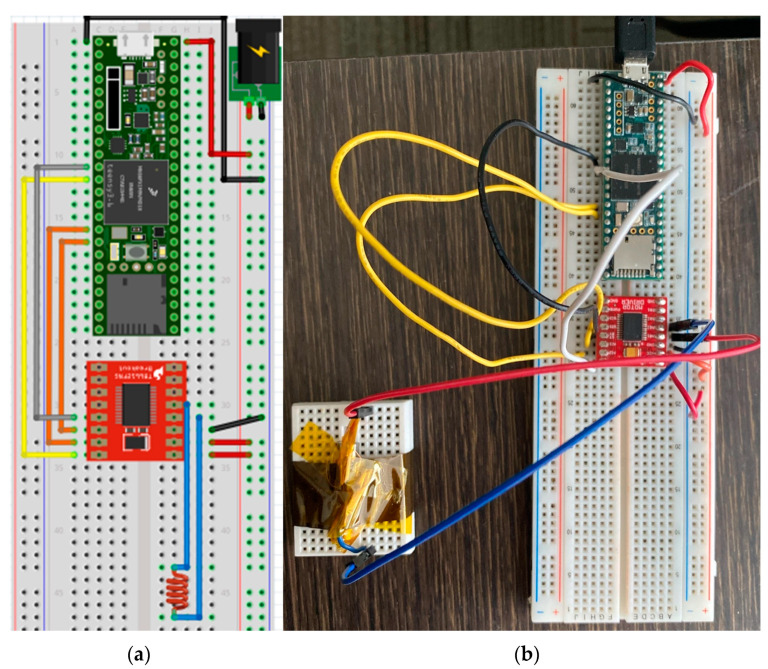
Circuit setup (**a**) Wire diagram of the ACS endurance testing; (**b**) Live picture of setup as tested.

The magnet coils were the next parts that were tested. There were two aspects of testing the endurance of the magnet coil: checking that the hardware could handle quick and frequent changes in PWM and checking that the system could stay on for long durations. The Teensy was connected to the H bridge which was connected to one torque coil for each of these tests. The IMU was not used and the PWM values were chosen randomly or periodically instead. The electrical diagram of the setup is shown in Figure 37.

##### Longevity Test

**Figure 38:**
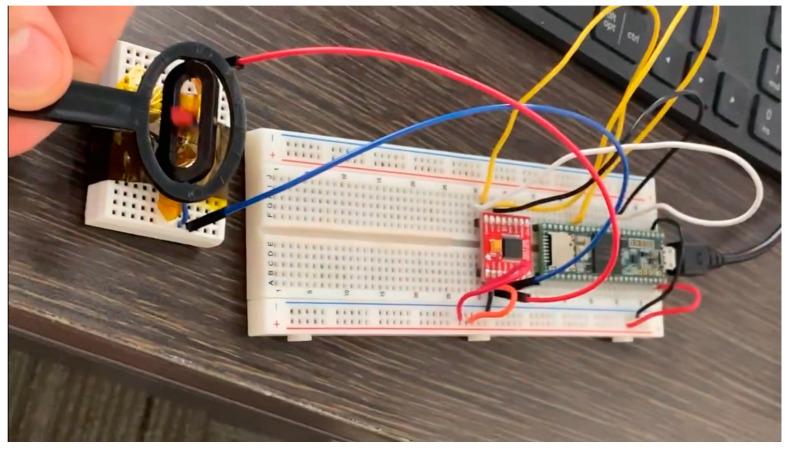
The gyrocompass measuring the magnetic field of the torque coil.

The system’s ability to run for a long time was tested in the longevity test. When the CubeSat leaves the ISS, the ACS system might need to run nonstop for several hours to stop tumbling and achieve its spin target. To check the reliability of the ACS subcomponents, especially the H bridge and the coil, a longevity test was conducted for two days. In this test, the PWM values were gradually increased from 0 to 255 for both polarities of the coil. Each PWM value for each current direction lasted for ten microseconds. The coil would change its polarity roughly every 2.5 s. A mechanical gyroscopic compass and timer were used to perform verification. Figure 38 below shows the verification of the functioning coil using the gyroscopic magnet.

The loop ran nonstop for two days. During that time, the magnetic field was measured using the gyroscopic magnet. The system remained active and precise for the whole period.

##### Fluctuation Test

The ACS system was tested to see how accurately it could respond to large and fast changes in the PWM value. This is because the controller might need to change the power drastically while detumbling due to the oscillating torque values from the Kane damper. It was observed that the flight computer had a typical reaction time of about 3 ms. So, a program was written to randomly pick a PWM value and current direction every 3 milliseconds. The magnetic field was then checked with the gyroscopic magnet. It seemed to change at an interval of a few milliseconds as a preliminary test. More testing was performed later using the IMU.

Also, longevity was tested again. Longevity tests were performed to see if the fluctuation over time would damage any of the hardware. The magnetic fields were measured every few hours. There was no noticeable change in the magnetic field strength, and the system worked similarly after two days. 

#### 4.3.7. Kane Damper Tuning

The ACS performance was improved by adjusting the Kane damper. A rough trial-and-error estimate was used to find the approximate convergence range of the CubeSat system. These initial values were also the starting point for the search algorithm discussed later. After choosing stable Id and c values, the controller was tested for robustness by changing the initial kinematic conditions. The controller showed robustness at different initial conditions. Lastly, a MATLAB^®^ algorithm was written to fine-tune the Kane damper.

The Kane damper was tuned with nested loops. The loops changed the scalar moment of inertia, Id, and the damping constant, c, of the damper. Since both values are directly proportional to the angular velocity, it was assumed that the surface plot would be smooth and continuous based on the plot made by Davide (Carabellese 39). The Kane tuner optimized computational time using a very coarse search. Instead of finding a precise array of damping constants and moments of inertia, the code would find a region with the fastest convergence time. For example, the parameters could be set up to find 25 values by increasing each variable (Id and c) by 5. A minimum rise time of the controller would be found for the coarse search. The nearby region would be zoomed in on and further searched. This convergence region would avoid wasting computational time finding precise solutions that are clearly not in the optimal region.

**Figure 39:**
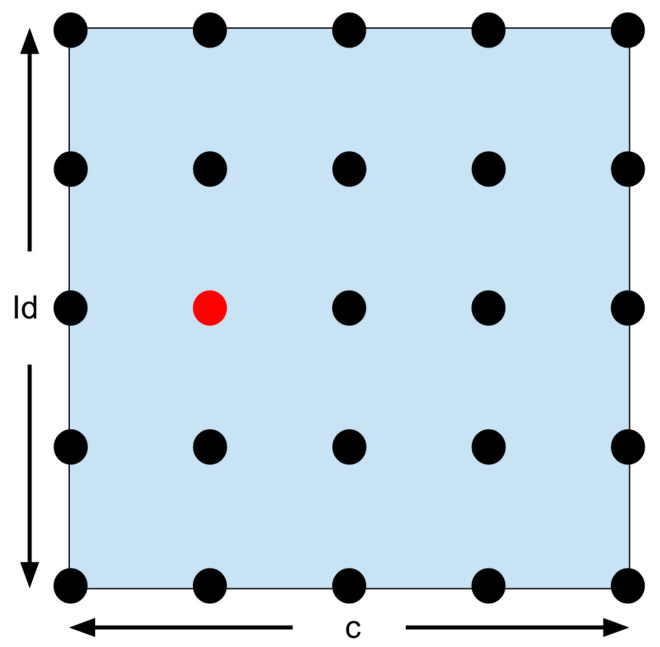
Confining search algorithm. The red dot symbolizes optimal result, within resolution of the search algorithm.

This confining search optimization technique is only possible because the surface plot is known to be smooth and continuous based on linearity. The plot referenced can be seen in Davide Carabellese’s thesis [19]. A visual representation of the first step of the confining search optimization formula can be seen below in Figure 39.

**Figure 40:**
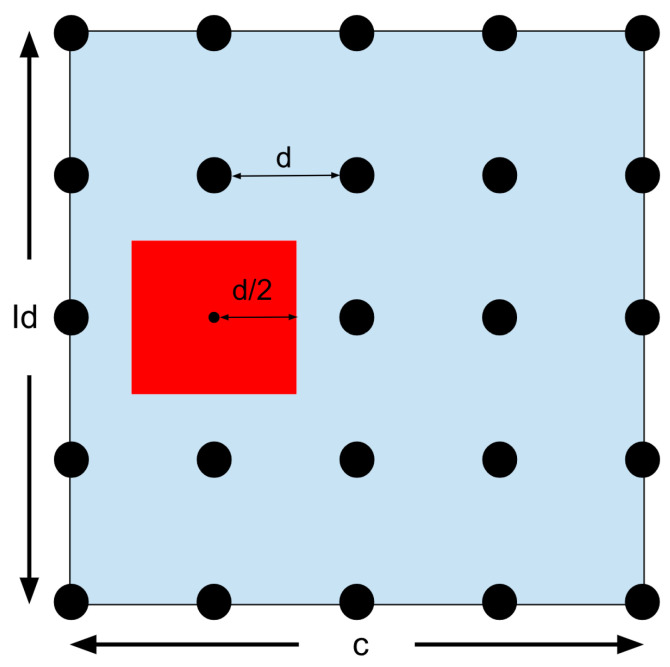
Zoomed-in search region. The red square symbolizes the region in which the optimal solution lies.

The 25 solutions are displayed as dots in Figure 39, with the best one in red. A zoom can be used to search more finely in the chosen area. The region closest to the red dot (half a deviation in each direction) becomes the new search region. The answer is bound to be in this region since both Id and c are related to the angular velocity. This means that the max and min are set to be half a deviation above or below the optimal solution. The updated search region is displayed below in Figure 40.

**Figure 41:**
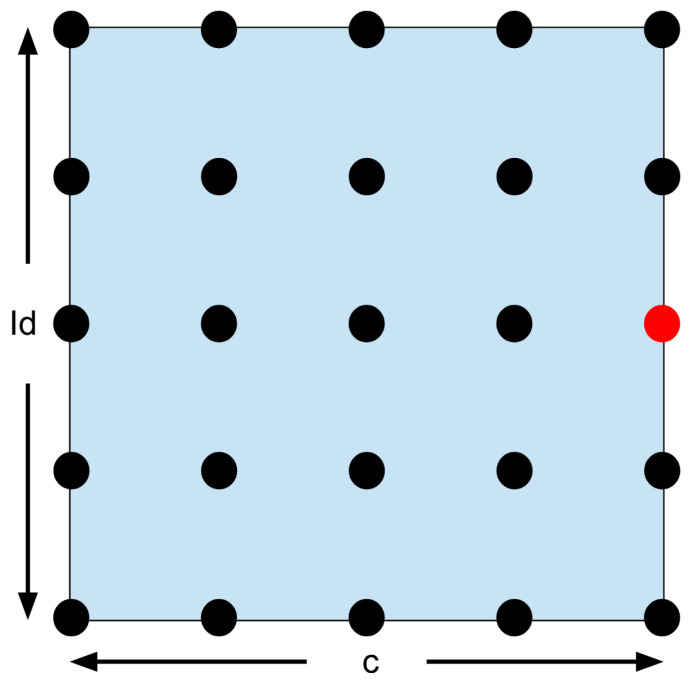
Optimal solution found on the border. This is shown by the red dot.

The zoom goes on until the required level of accuracy is reached. But if the best solution is on the edge of the search area, the problem becomes more complicated. Look at Figure 41.

**Figure 42:**
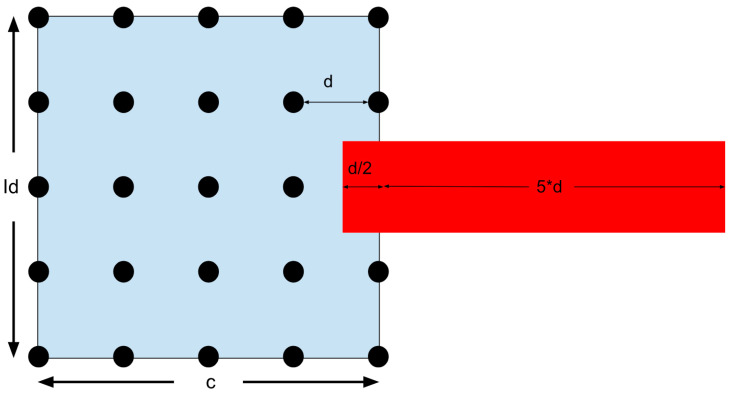
Borderline search region with autonomous search region expansion. The red square symbolizes a growth in the search region in order to search beyond the boarder.

In this situation, the answer might be right at the edge, or it might be outside of the search area. The MATLAB^®^ script will show a popup window for the operator to choose. The program can either keep searching along the edge or it can independently enlarge its search area. If independently enlarged, the search area will increase by the same interval as originally set. But it will also keep a half unit inside the old area in case the operator is mistaken and the answer is near the edge. See Figure 42.

Because the zoom did not succeed, the zoom factor will not increase by one.

**Figure 43:**
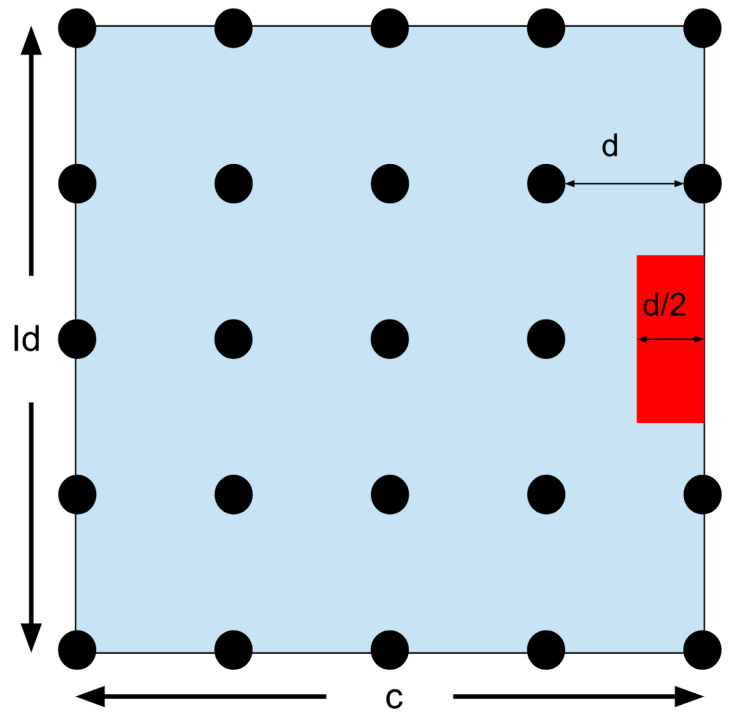
Borderline search region without expansion. The red square symbolizes a decision not to expand the search region, but to confirm a boarder result by searching within the convergence field.

The operator might pick the other option if they must enlarge the search region or if they think that the best solution is inside the original definition. If they choose to keep the original search parameters, they will maintain the same search area and keep zooming in on the edge. See Figure 43.

**Figure 44:**
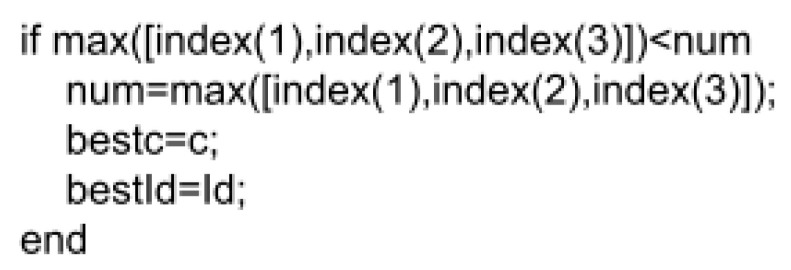
Pseudocode to find optimal Id and c.

Figure 43 shows that the zoom will follow the border. The result was obtained by finding the three indices where each of the three angular velocities X, Y, and Z met. The index of the longest settling time of the X, Y, and Z angular velocities was noted. The note was made because the ACS would be seen as converged when all the desired angular velocities had been achieved. The index of total settling time was contrasted as the algorithm increased Id and c. When a lower index was found, it would take over the previous one and note the respective Id and c values. Figure 44 below presents a pseudocode that illustrates the logic that was mentioned.

**Figure 45:**
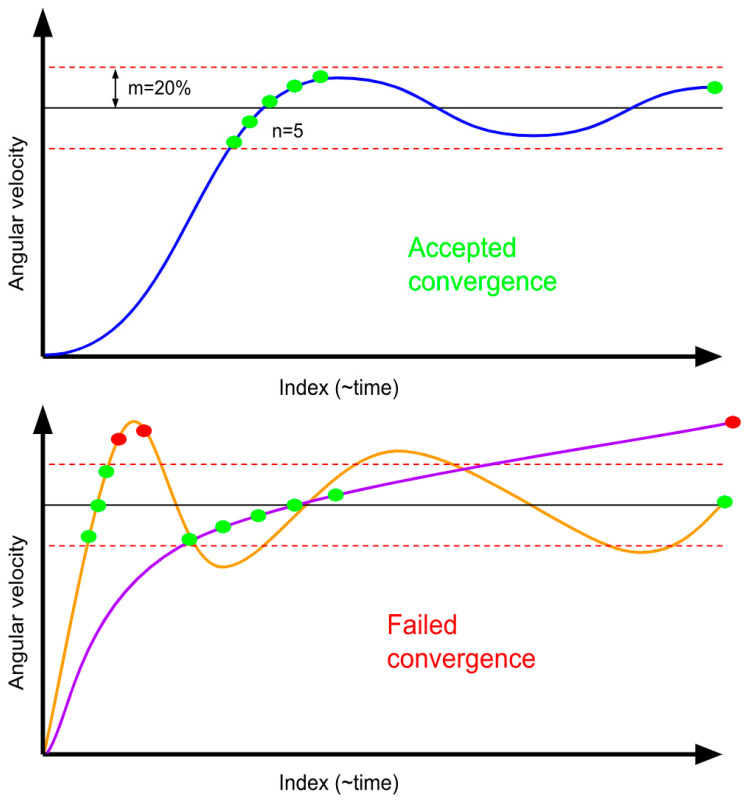
Convergence criteria. *n* number of points within an m% region of convergence, and the endpoint must lie in the region of convergence. (*n* = 5 and *m* = 10).

The tuner’s performance was measured by the time it took for the system to reach the target conditions. The time of convergence was determined by finding the point where the average of the previous “*n*” values was within a “*m*” percent range of the desired angular speeds. Moreover, to ensure the stability of the solution, the final value was checked to see if it stayed in the convergence range. For the tuner, 5 and 20 were selected as the values of “*n*” and “*m*”. See Figure 45 below.

The MATLAB^®^ code was executed at a zoom level of 3. The MATLAB^®^ optimization code produced the best values for Id and c, which are shown in Table 8 below. Using these values, the controller achieved its convergence in 21 min. A 21 min convergence time satisfies requirement 2.1.

#### 4.3.8. Tuning the IMU against Electronic Induced Offsets

The IMU measures the earth’s magnetic field, so it needs to avoid being affected by the CubeSat’s own magnetic fields. To measure the earth’s magnetic field correctly, the IMU needs to be calibrated to remove the impact of the CubeSat dipoles. The CubeSat produces two types of magnetic offsets: hard iron offsets and soft iron offsets. The hard offsets are the magnetic fields that come from the electronics onboard. The hard offsets are a constant value that can just be subtracted from the IMU reading. The soft iron offsets, however, are more complicated because they vary depending on the controller. The offsets come from the magnetic fields created by the magnetic torquers.

**Figure 46:**
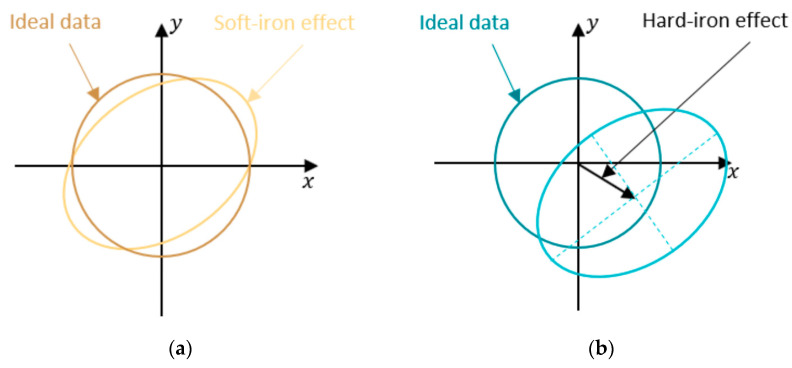
Effects of hard iron and soft iron offsets. (**a**) Skew matrix, and (**b**) displacement vector.

To explain the effects of these better, the hard iron offsets are displacement vectors. The offsets shift the three-dimensional readings linearly. The soft iron offsets, on the other hand, are distorted by a transformation matrix that depends on the power of each magnetic torque coil. See Figure 46 below.

Moreover, the CubeSat’s temperature had fairly big impacts on the readings from the magnetometer. Our model would also need to consider these effects.

##### Soft Iron Offsets

The magnetic torque coils produce soft iron offsets that will taint the IMU readings. The soft iron offsets differ from hard iron in that they fluctuate as a function of the power of the system. Each of the three torque coils will affect the magnetometer reading independently based on the current through the coil and its relative position with respect to the magnetometer. The controller constantly changes the current in each of the three coils, and the magnetic fields of the torque coils cause offsets that need to therefore be continuously re-evaluated. These values are then taken away from the IMU magnetometer reading in real time. However, it turned out that current was not the only thing that influenced the magnetic field through the coils. Experimentation and system verification showed that the magnetic offsets vary as the battery drains because of the lowered voltage. So, a model was created that would give soft iron offset values based on the current input to the X, Y, and Z coils and the current battery voltage.

##### PWM Offsets

To achieve the pulse width modulation (PWM) offsets, data were gathered for how the magnetometer’s magnetic field changed in the X, Y, and Z directions for each of the three coils. Each coil creates a three-dimensional offset vector because there is a displacement vector between the IMU reference frame and the coil’s reference frame. With three coils, nine different offset values were obtained (three coils with three dimensions each). These were added together, giving an overall three-dimensional offset vector. The data collection script was sent to the CubeSat with an instruction to turn the magnet coils on for one second. Magnetometer readings were taken every 0.0015 s. Every ten readings were averaged and shown on the serial monitor. Using a serial monitor to .txt file converter called PuTTY, the data were saved and later copied into an Excel sheet. A marker was also shown to clearly indicate the interval when the coil was on. Once imported into Excel, these measurements were then averaged again, before and after the markers. The averages while the coil is on in the X, Y, and Z directions were compared to the same respective averages while the coil is off. The resulting vector represents a three-dimensional offset for the coil tested.

**Figure 47:**
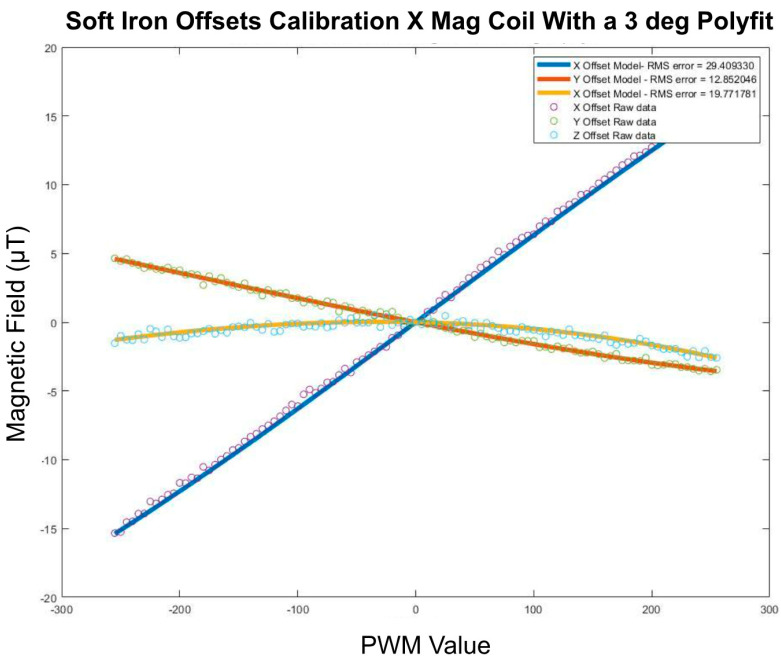
X Coil 3-degree polyfit IMU offsets.

During the experiment, one coil was turned on at a time, beginning with the X coil. The PWM value was increased by increments of five, starting from 255 to 0 in the forward and reverse direction. The offsets were measured as a three-dimensional vector, a function of the input PWM level. After making a matrix with offsets over the PWM range, the raw data were loaded into MATLAB^®^. A script was written to help an operator find the order of the best-fit polynomial. The line of best fit was then plotted on top of the raw data. To assess the goodness of fit, a root mean squared error (RMS) was calculated and displayed in the legend. The second and third-degree polyfit models had the highest marginal return. See Figure 47 for a three-degree polyfit below.

**Figure 48:**
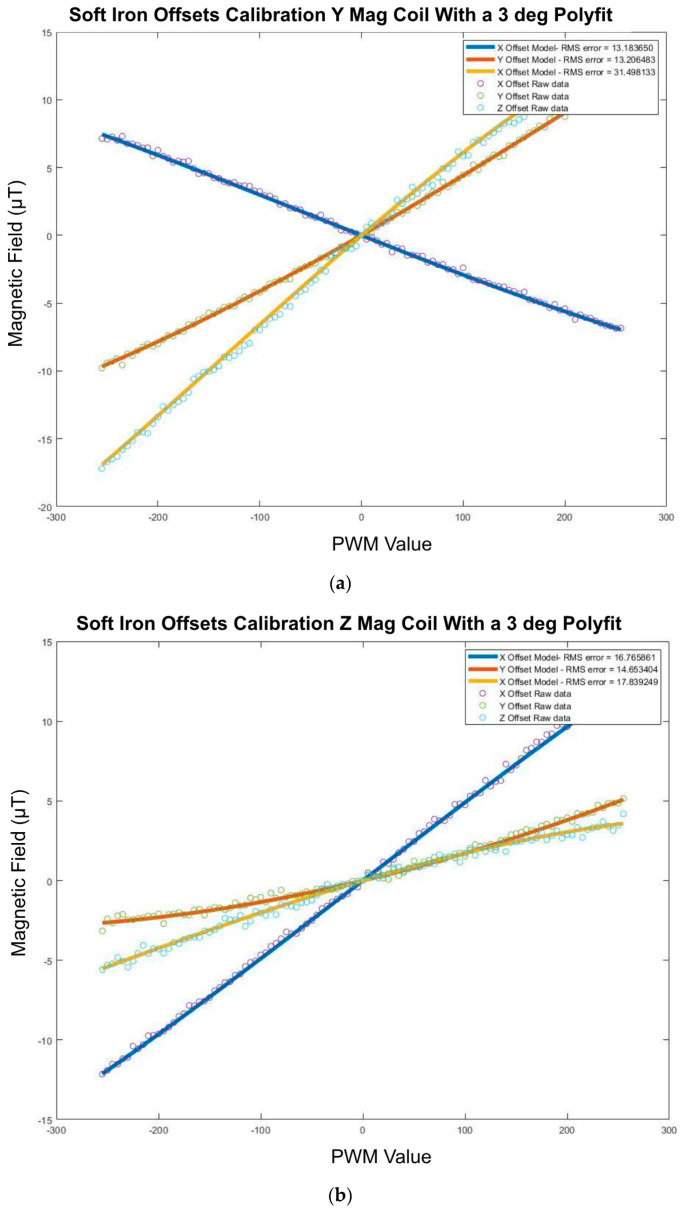
(**a**) Y coil 3-degree polyfit IMU offsets. (**b**) Z coil 3-degree polyfit IMU offsets.

Similarly, the same method was applied to increase the PWM values in the Y and the Z magnetic coils. Comparable graphs were created. See Figure 48.

The above nine polyfits show how each of the three magnet coils affects each of the three coordinate axes. By adding all three magnet coil effects, these equations could be combined into just three: total X offset, total Y offset, and total Z offset. Each of these three final equations would depend on all three PWM value inputs to each of the magnetic torque coils. To adjust the curves above properly, the CubeSat battery voltage was measured. Comparing it to the battery charge would give a reference for the voltage correction. The CubeSat had a full battery (4.2 V) when the PWM tests were performed.

##### Battery Voltage Offset Correction

We noticed that the soft iron offsets decreased as the CubeSat’s battery voltage went down. The battery voltage could vary from 4.2 to 3.7 volts over the charge cycle of the battery. The ACS would automatically shut down to save power if the battery went below 3.7 V, so we did not consider lower voltages. The change in voltage from a full to an empty battery did not affect the magnetic field much. The differences were 1–2.5 times the resolution error of the IMU magnetometer. We first thought of a linear subtraction because it was fast and easy. But we knew that the PWM and offset correlation was not linear, so this method seemed too simple. We did not need the extra precision, but we calculated a correction coefficient instead. We collected data by connecting the CubeSat to a variable power supply and changing the voltage from 4.2 to 3.7 volts by 0.1 volts each time. We did this for each of the three coils, and the changes in offsets were measured along all three coordinate axes by the IMU. The PWM value of each coil was set to 255. We did nine experiments in total. The average change in the offsets from 4.2 to 3.7 volts was recorded for all nine cases. The value was then normalized. The normalization was performed to find the fraction of change in the offsets compared to full battery conditions. Since the experiments were performed at a PWM value of 255, normalizing against the 4.2 volt 255 PWM value would give the percentage of change in the offsets at 3.7 volts. This value shows the proportional difference in the magnetic offsets between a full battery and 3.7 volts. We programmed a linear gradient that could use any input voltage and correct the change in offsets accordingly. Subtracting the value from one would show the factor by which the polyfit would have to flatten, the correlation coefficient ($C_{Correlation\;}$). See Equation (12) below. Here $offset(PWM\left(255\right)\;,battVolts)$ represents an interpolated instantaneous offset (based on battery voltage), and $offset(PWM\left(255\right)\;,battVolts)$) represents the offset at full battery (which is used to normalize).

(12) $$C_{Correlation\;}=1-\frac{offset\left(PWM\left(255\right),\;\;4.2\;V\;\right)-offset(PWM\left(255\right)\;,battVolts)\;}{offset\left(PWM\left(255\right),\;\;4.2\;V\;\right)}$$

This equation uses the “offset” function to achieve the magnetic field offsets at the conditions in the parenthesis. The variable battVolts is the current battery voltage. Using Equation (12), all nine correlation coefficients were calculated. The resulting coefficients were then multiplied by each of the corresponding nine polyfits based on the PWM values (explained in the previous section). This method was accurate both experimentally and theoretically. If the battery is full, the correlation coefficient is 1, and nothing changes. As the battery drains, the coefficient is adjusted according to the difference in the offsets measured experimentally. If the battery voltage was zero, each of the polyfits would be zero, as expected. The polyfit approach is therefore much more reliable and robust than a simple linear subtraction. To test the soft iron solution, offsets were predicted using the model with random PWM inputs. The IMU offsets matched the predictions. Therefore, the battery model satisfied requirement 6.1 in Table 5.

Hard iron offsets are the effects that the electronics on the CubeSat have on the magnetometer readings. These are static magnetic fields that exist whenever the satellite is on. To find the hard iron offsets, a procedure from Adafruit was followed (LeBlanc-Williams, Adafruit, 1 June 2020). A detailed procedure can be found in the references. To find the hard iron offsets, the IMU had to be on. The IMU was started using a sample Arduino script from the Adafruit sensor library. Running the script turned on both the gyroscope and the accelerometer. With both, the IMU could know how it was being moved relative to the gravitational field. Also, the magnetometer was on to sense the constant offsets surrounding the sensor. With these sensors active, the resulting values could be read by other software. The software used was called “Motion Sensor Calibration Tool”. Downloading this tool allowed for the hard iron offsets to be calibrated by rotating the CubeSat around the IMU. Datapoints were collected as the CubeSat was rotated. A trajectory path was drawn with each rotation. Once enough of the surface area had been drawn, offset values were shown. Figure 48 below shows a snapshot of the process.

**Figure 49:**
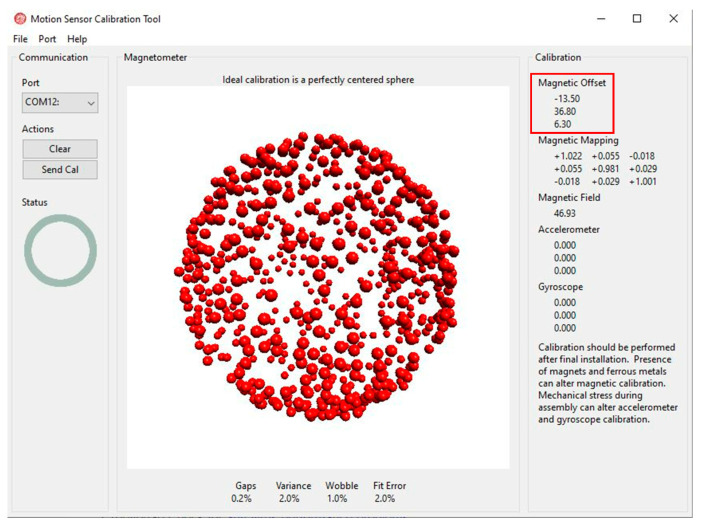
Motion sensor calibration tool shows hard iron offsets.

Figure 49 shows a clear contour that demonstrates the rotational field of the IMU. The upper right-hand corner displays the hard iron magnetic offsets. The experiment was repeated five times, and the values were averaged. Table 9 below shows the results of each trial.

The motion sensor calibration tool gave very accurate and consistent measurements, as shown in Table 9. These values met the requirement 6.0 in Table 5. The soft iron offsets were added to these values to achieve the total iron offsets.

#### 4.3.9. Temperature Offset

**Figure 50:**
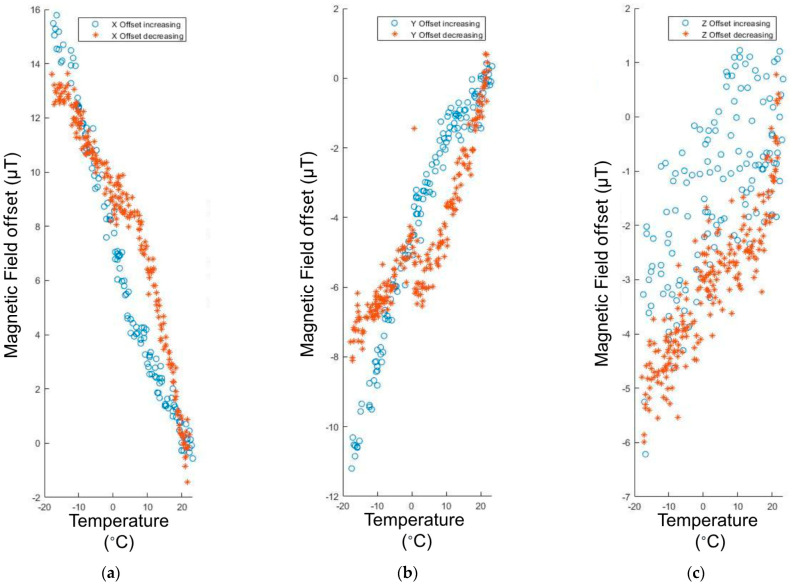
Negligible hysteresis error in temperature offset. (**a**) x axis magnetic field offsets as a function of temperature increasing (blue) and decreasing (red); (**b**) y axis magnetic field offsets; (**c**) z axis magnetic field offsets.

The temperature of the CubeSat was another correction factor that was considered. It was observed that the IMU’s offset reading goes up as the CubeSat’s temperature goes down. This correlation was expected to be due to lower resistance in the IMU. Experimental data were gathered in a suitable temperature range that the CubeSat might encounter in LEO. A case study showed that CubeSats in LEO often have temperatures ranging from −5 degrees Fahrenheit in the Earth’s shadow to 130 degrees Fahrenheit when exposed to the sun’s light (Dinh 11). Ambient conditions tend to vary a lot more. But, because of the satellite’s spin, residual heat, and waste heat from the electronics, the temperature is more stable. To test for hysteresis, the experiment was divided into two different parts. In the first experiment, the CubeSat began at room temperature, 73 degrees Fahrenheit. It was wrapped in an ESD-safe bag and moved into a cooler with dry ice to observe the effects of cooling. The temperature and magnetic field were recorded. Just like the procedure used when calculating soft iron offsets, the temperature and magnetometer values were averaged over ten values. However, data was only printed to the serial monitor every ten seconds. A bigger timestep was used to fit the longer characteristic time of the test and to avoid too much data. The magnetic field strength was measured until a temperature of −18 degrees Fahrenheit was reached. The next test was performed starting in the dry ice and moving the CubeSat under heat lamps. These heat lamps were meant to imitate solar radiation. The CubeSat was placed on an ESD bench and allowed to heat from −18 deg to 134 deg Fahrenheit. Again, the magnetic field was recorded throughout the test process. A continuous dataset of magnetic fields from low to high temperatures was recorded. To evaluate the offsets, the magnetic field at ambient temperature was subtracted from both datasets. The sets were compared to each other to check for hysteresis errors. Please see Figure 50.

**Figure 51:**
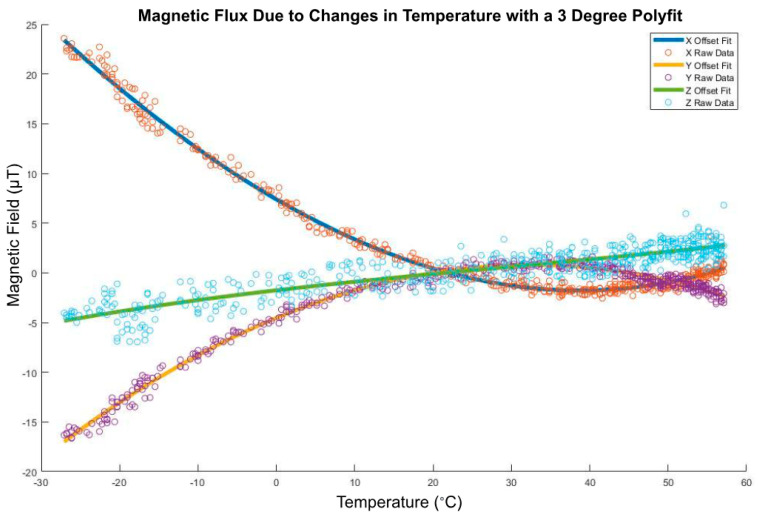
Magnetic flux due to changes in temperature with a three-degree polyfit.

After examining the data, the error was determined to be insignificant compared to the IMU’s resolution. The dataset with increasing temperature was chosen because it covered the whole range of interest from −18 deg Fahrenheit to 134 deg Fahrenheit. A polyfit was applied to the dataset with an increasing temperature. The best degree of fit was found by gradually increasing the fit order and looking at the small diminishing returns in terms of the RMS error. A third-degree polynomial was the most effective. See Figure 51.

The temperature model was validated in a similar way to the soft iron model. The experimental values matched the simulated ones closely. The resemblance indicated that the temperature model satisfied requirement 6.2 in Table 5. The polyfit equations shown in Figure 51 above could be combined with the soft and hard iron offsets to obtain the final offsets of the IMU magnetometer.

## 5. Conclusions

The work performed for the Alpha mission used a systems approach to improve the TRL of the ChipSat RF system and the ACS system. The progress followed the standard “Vee” diagram format. Requirements were defined throughout the design lifecycle, by breaking down the concept into subsystem and component solutions. Requirements were traced into experimental testing, where verification and validation confirmed prototype functionality.

Using interface and network diagrams, the system was divided into the system and component levels. At these levels, the traceability of resources and signals was examined, and requirements between systems were established. The theory of the model was tested by verifying that each subsystem met its intended use. Finally, full system validation could be performed through mission testing.

During development, confidence tests were performed to continually verify the functionality of the solution. The RF system was already validated for Alpha by Dr. Manchester [42] and Dr. Adams [15]. Building on their work, validation of an RTOS-based ChipSat code with a signal ready for matched filtering and FEC was achieved. RF transmission from a CC1310 microcontroller was tested during a balloon launch. Moreover, ground station connections could be established during balloon testing. Signals could be received using both a software-defined radio (RTL-SDR) and a receiver network (TinyGS). In addition, these signals could be FEC post-demodulation. For the ACS system, both validation and verification of the system were performed.

### Discussion of the Results

The PD controller method was proven valid by comparing the solution computationally to other industry-standard controllers. Tested control algorithms included the P+V controller, the P + V Double Integrator Controller, the Double Integrator Gain Tuning Controller, the 2DOF Feed Forward Controller, the P + V Control Law Inversion Controller, the Open Loop Guidance Controller, and the Real-Time Optimal Controller. The PD controller had the lowest quadratic cost while maintaining a robust rise time (w.r.t. the computational timestep). Validation of the controller was performed through experimentation, including moment of inertia testing, amplification factor testing, and endurance tests of the hardware. These tests showed the functionality of the respective subsystems. Once testing was performed, an optimization code was written, and the optimized result showed the fastest convergence within a defined search region. The optimal Kane Damper moment of inertia and damping constant were 0.07 and 0.0025, respectively. Finally, tuning was performed for the IMU. A model was created to adjust for hard iron offsets, soft ion offsets, changing battery voltage, and temperature changes. Each regression was superimposed into one model. The resulting code was tested at arbitrary values. By keeping a systems perspective throughout the satellite lifecycle, the requirements for the developed systems were met.

## Figures and Tables

**Table 1:** System top-level black box interfaces.

Variable/Acronym	Definition	Variable/Acronym	Definition
F1	Solar panel	F3	Thermistor
F2	Ambient light sensor	F5	Gyroscope
F6	Malfunctions/damage	F8	RF transceiver
F7	Torque coils	F9	Dissipated light
F10	Dissipated heat		

**Table 2:** Immediate subsystem.

Variable/Acronym	Definition
F4	IMU

**Table 3:** Full system breakdown.

Variable/Acronym	Definition	Variable/Acronym	Definition
F11–F14	Analog to digital converter	F15	PWM Modulator
F16	Data Packager		

**Table 4:** Requirement table for the ChipSat RF subsystem.

ID	Requirement	ID	Requirement
1.0	The systems SHALL communicate over RF	5.0	The system SHALL add a preamble
2.0	The system SHALL capture IMU data in a packet	5.1	The preamble SHALL consist of 4 8-bit barker codes
2.1	The system SHALL have 16-bit data	6.0	The system SHALL apply matched filtering
2.2	The system SHALL collect magnetometer, accelerometer, and gyroscope data in 3D space	7.0	The signal SHALL be received by a ground station
3.0	The system SHALL be frequency modulated	7.1	The signal SHALL be demodulated
4.0	The system SHALL forward error correct (FEC) the data		

**Table 5:** Requirement table for the CubeSat ACS subsystem.

ID	Requirement	ID	Requirement
1.0	The system SHALL detumble to 10% of the initial angular velocity in the x and y direction	5.0	The Teensy flight computer SHALL be able to handle the ACS computations
2.0	The z angular velocity SHALL spin stabilize within 10% of omega_final = [0 0 1] rad/s	6.0	The system SHALL calibrate the IMU against hard iron offsets caused by internal electronics
2.1	The system SHALL spin stabilize within 8 hours	6.1	The system SHALL calibrate the IMU against soft iron offsets caused by actuators
3.0	The system SHALL point its *z* axis tangent to the Earth’s surface a majority of the time	6.2	The system SHALL calibrate the IMU against temperature offset effects
4.0	The controller SHALL not use more than 0.9 watts (0.2 amps at 4.2–3.7 volts)		

**Table 6:** Characteristics of optimized industry standard controllers.

	Quadratic Cost	θ End Point Error	θ Standard Deviation	Rise Time
PD controller	1.309573	9.28393 × 10^−2^	4.857353 × 10^−2^	8.57 × 10^−1^
P + V Controller	55.709082	8.239661 × 10^−3^	1.061509 × 10^−2^	3.90 × 10^−1^
P + V Double Integrator	2.544054	1.216665 × 10^−1^	1.044603 × 10^−2^	N/A *
Double Integrator gain tuning	6.579885	1.087292 × 10^−1^	1.085613 × 10^−2^	7.80 × 10^−1^
2 DOF feed forward	6.147382	2.911532 × 10^−2^	1.115621 × 10^−2^	8.00 × 10^−1^
P + V Control Law Inversion	6.127791	2.929454 × 10^−2^	1.106685 × 10^−2^	8.00 × 10^−1^
Open loop guidance (DQC)	6.181200	5.775071 × 10^−3^	5.761962 × 10^−2^	8.00 × 10^−1^
Real-Time Optimal Controller	6.169038	1.865604 × 10^−5^	2.710996 × 10^−3^	8.10 × 10^−1^

* does not have a formal rise time.

**Table 7:** Robustness of the controller to varying sample rates.

Sample Rates (Seconds)	Rise Time (Seconds)
0.1	8.67 × 10^−1^
0.01	8.29 × 10^−1^
0.001	8.20 × 10^−1^

**Table 8:** Final Id and c calculated to optimize the CubeSat Kane damper.

Id	c
0.07	0.0025

**Table 9:** Hard iron offsets recorded to calibrate the IMU of static EM radiation from onboard electronics.

Trial	X Offset	Y Offset	Z Offset
1	−14.80	35.81	7.13
2	−13.10	36.68	7.04
3	−13.50	36.80	6.30
4	−13.70	34.78	7.46
5	−13.15	36.22	6.71
avg	−13.65	36.058	6.928

## Data Availability

The original contributions presented in the study are included in the article, further inquiries can be directed to the corresponding author.
